# A Transformers Analysis Pipeline (TrAP) to Evaluate LINE-1 RNA Sequence Content

**DOI:** 10.3390/ijms27219762

**Published:** 2026-10-30

**Authors:** Andres D. Chamorro-Parejo, Kenneth S. Ramos

**Affiliations:** Center for Genomic and Precision Medicine, Texas A&M Institute of Biosciences and Technology, Texas Medical Center, Houston, TX 77030, USA

**Keywords:** LINE-1 retrotransposon, regulatory RNAs, RNA monitoring techniques, transposable element quantification, short-read RNA sequencing, transformer models, k-mer tokenization

## Abstract

LINE-1 RNA has emerged as a potential regulator of chromatin organization and gene expression, as well as a candidate readout of cellular and disease-associated states. Accurate measurement of LINE-1 transcript abundance is therefore important for evaluating its regulatory, biomarker, and potential therapeutic relevance. Measurement is challenging in short-read RNA sequencing because similar LINE-1 loci produce ambiguous, multi-mapping reads and LINE-1-associated signal can arise from passive as well as autonomous transcription. We developed the Transformers Analysis Pipeline (TrAP), an alignment-free ALBERT-based workflow that classifies short RNA-seq reads as L1HS, L1PA, or non-LINE-1 before abundance estimation, converting overlapping k-mers to integer tokens and integrating predictions with Salmon- or expectation–maximization-based quantification. TrAP was evaluated using synthetic LINE-1 insertion datasets and HEK293 short-read RNA-seq benchmarked against 5′ rapid amplification of cDNA end (5′RACE) long-read measurements. AlbertSalmon agreed most closely with the synthetic insertion levels, ranking first in all insertion levels and deletion-probability strata. Agreement with the 5′RACE reference was also highest, although the locus-level gain there was only incremental. High-attribution k-mers were modestly over-represented within functional ORF2 coding regions rather than being uniformly distributed across LINE-1. These findings support TrAP as a transformer-assisted framework for improving LINE-1 quantification in short-read RNA-seq datasets, thereby supporting future regulatory and biomarker studies.

## 1. Introduction

Long Interspersed Element-1 (LINE-1), also commonly referred to as Long Interspersed Nuclear Element-1, is a non-Long Terminal Repeat (non-LTR) autonomous retrotransposon found in the human genome. The LINE-1 family of retroelements has been implicated in genome evolution, DNA damage and repair, and chromatin remodeling [1]. Beyond these element-level activities, LINE-1 transcripts are increasingly recognized as regulatory RNA molecules. LINE-1 RNA partners with nucleolin to regulate ribosomal RNA synthesis and stem-cell identity [2]. LINE-1 elements are normally repressed but can become reactivated in cancer, where they are linked to genome instability, altered gene regulation, chromatin remodeling, tumor evolution, and cellular plasticity [3]. Transcription from individual LINE-1 loci activates gene expression over long genomic distances [4]. This places LINE-1 within the broader class of regulatory non-coding RNAs, alongside long non-coding RNAs (lncRNAs), whose expression patterns distinguish transformed from non-transformed lung epithelial cells and contribute to the cellular heterogeneity of lung tumors [5]. These observations place LINE-1 RNA abundance among the quantities of biological interest. A full-length retroelement consists of a 5′-untranslated region (5′UTR), two non-overlapping open reading frames (ORF1 and ORF2), and a 3′UTR. Although ORF1 and ORF2 encode the principal proteins required for retrotransposition, primate LINE-1 elements also contain ORF0 within the 5′UTR in the antisense orientation [6]. The present study analyzed human-specific LINE-1 (L1HS) and primate-associated LINE-1 (L1PA) sequences. The consensus annotation treated the 5′UTR as a single region. LINE-1 is epigenetically silenced in most somatic cells but can be reactivated to participate in cancer initiation and progression [7,8]. LINE-1 assembly and annotation remain challenging when using sequencing technologies that generate reads significantly shorter than 6 kb, the average length of LINE-1 [9]. The highly repetitive nature of LINE-1 makes it particularly difficult to assign short reads to specific genomic loci and to resolve closely related LINE-1 copies and subfamilies, including L1HS and L1PA. Thus, the primary computational limitation arises from the ambiguity arising from intra-family sequence similarity, fragmented LINE-1 copies, and the multi-mapping behavior of short reads. These properties complicate the identification and quantification of LINE-1-derived signals in short-read sequencing data.

Transcript-level measurements reflect LINE-1 RNA production, rather than the potential regulatory activity of LINE-1 sequences as DNA elements [4]. However, not every LINE-1-aligned signal detected by short-read RNA sequencing represents autonomous LINE-1 transcription. A substantial fraction of the apparent LINE-1 expression in bulk RNA-sequencing libraries may instead arise passively from intronic LINE-1 copies co-transcribed with host pre-mRNAs, transcriptional read-through, or incompletely spliced RNA, rather than from transcription initiated at the LINE-1 5′UTR [10,11]. LINE-1-derived transcripts occur across a spectrum of forms rather than two discrete classes. These include full-length transcripts initiated at the 5′UTR sense promoter, chimeric transcripts driven by the LINE-1 antisense promoter, 5′-truncated and internally initiated species, and LINE-1 sequences carried passively within host pre-mRNA introns. Several non-autonomous forms, particularly antisense-promoter-driven chimeric transcripts, are well documented to exert regulatory effects in human cells [12,13,14]. Interpreting LINE-1-derived signal therefore requires, as a first step, resolving the LINE-1 subfamily from which a given read derives, as addressed by TrAP, independently of the locus that produced it. This distinction connects the computational challenge directly to the underlying biology. Any claim that LINE-1 RNA functions as a regulator, or that its abundance reflects a particular cellular state, requires evidence that the measured signal arises from the transcription of LINE-1 elements rather than from incidental transcription through LINE-1 sequences. Separating reads that support transcription initiated at a LINE-1 promoter from those generated by passive co-transcription is one distinction within this spectrum, and resolving it extends beyond multi-mapping ambiguity. It is a prerequisite for ensuring that the resulting abundance estimates carry the biological meaning attributed to them.

Existing approaches meet these requirements only in part. Conventional LINE-1 detection and quantification methods include reference-based annotation, repeat-aware RNA-seq quantification, and pattern matching [15,16,17,18]. Although contemporary RepeatMasker workflows incorporating Dfam and profile hidden Markov models have substantially improved repeat annotation [19], these advances do not fully resolve the specific problem addressed here: quantifying LINE-1-derived transcriptional signal from short-read RNA-seq data. Individual LINE-1 copies often undergo internal deletions that truncate their DNA sequence and compromise functionality, while segmental duplications generate multiple copies of LINE-1-containing regions, adding complexity to accurate identification and quantification. Transposable Element (TE)-aware RNA-seq quantifiers address multi-mapped reads through counting or probabilistic reassignment. TEtranscripts estimates TE expression primarily at the family or subfamily level, whereas L1EM uses expectation–maximization to estimate locus-specific LINE-1 abundance. Salmon provides lightweight probabilistic transcript quantification that can be adapted to LINE-1 reference annotations, while HTSeq provides a conventional feature-counting baseline. These approaches improve repeat-aware quantification but retain assumptions associated with reference annotation, alignment or pseudo-alignment, and probabilistic assignment of ambiguous reads.

Pattern-matching methods provide a complementary strategy for genomic LINE-1 detection. The LINE-1 Pattern Detection approach uses ordered k-mer probes and their relative positions to identify intact and truncated LINE-1 copies with high precision, avoiding the heuristic extension step used by conventional seed-and-extend alignment [20]. However, this approach is designed primarily for assembled genomic sequences rather than direct read-level classification of RNA-seq data. Alignment-free machine-learning methods instead classify sequences from intrinsic characteristics such as k-mer composition, but their performance depends on the quality and representativeness of the training data and does not necessarily provide locus-level resolution. Together, these limitations motivate a read-level framework that can distinguish LINE-1-derived reads before abundance estimation without replacing established annotation or quantification tools.

Machine Learning (ML) methods have been increasingly applied to genomic sequence analysis, progressing from convolutional architectures such as DeepBind and DeepSEA to transformer-based approaches such as DeepConsensus and Evo [21,22,23,24]. Convolutional models identify informative local sequence patterns, whereas transformer architectures use self-attention to model contextual relationships among sequence-derived tokens within a defined input window. Although transformer architectures originated from natural-language applications, their use here constitutes genomic sequence modeling rather than natural-language processing.

Alternative approaches based on alignment-free sequence representations and ML classification have also received increasing attention [25]. By operating on sequence-derived features such as k-mer composition, these approaches can reduce dependence on explicit read-to-reference alignment, although their computational requirements and generalizability depend on the representation, model architecture, and training data [26,27].

The Transformers Analysis Pipeline (TrAP) uses A Lite BERT (ALBERT), a parameter-efficient transformer architecture derived from Bidirectional Encoder Representations from Transformers (BERT) that reduces model complexity through factorized embedding parameterization and cross-layer parameter sharing while retaining contextual sequence modeling. ALBERT was selected to support read-level classification with fewer trainable parameters than a comparably configured BERT model. Within TrAP, the classifier processes k-mer-tokenized RNA-seq reads and assigns them to L1HS, L1PA, or non-LINE-1 classes before downstream abundance estimation.

Here, we present TrAP, an end-to-end transformer-assisted framework for detecting and quantifying LINE-1 sequence content in short-read RNA-seq data. The effectiveness of transformer-based genomic models depends on how nucleotide sequences are represented before model input. In the present study, each read was decomposed into overlapping k-mers, and each observed k-mer was mapped one-to-one to a unique integer identifier in a fixed vocabulary. This tokenization strategy determined the range over which increasing *k* produced diminishing gains in sequence-complexity signal within the transcript-derived training corpus. This approach was informed by the work of Wu et al. (2009), who used cumulative relative entropy and relative sequence divergence to guide empirical k-mer length selection in phylogenetic inference [28].

The innovation of TrAP lies not in using ALBERT as a standalone classifier but rather its integration into a complete LINE-1 analysis workflow that combines k-mer/tokenization optimization, read-level multi-class classification, and downstream abundance estimation. The classifier assigns reads to L1HS, L1PA, or non-LINE-1 classes and uses these predictions in two downstream strategies: Albert with Salmon (AlbertSalmon) and Albert with expectation–maximization algorithm (AlbertEM). The workflow was benchmarked using synthetic LINE-1 insertion datasets with controlled mutation, deletion, truncation, and insertion parameters and was further evaluated in HEK293 short-read RNA-seq data using 5′ rapid amplification of cDNA end (5′RACE) long-read sequencing as an orthogonal reference. The classifier was trained using synthetic transcript-derived reads and RepeatMasker-derived labels, which provided a reproducible supervised training scaffold rather than independent biological ground truth. Moreover, TrAP performed read-level classification and relied on downstream estimators for locus-level abundance.

We conclude that read-level transformer classification, coupled with established abundance estimators, provides a complementary alignment-free strategy for evaluating LINE-1 sequence content in short-read RNA-seq data. AlbertSalmon was the preferred TrAP implementation as it shows the strongest agreement using synthetic insertion data and 5′RACE reference measurements. We also found that the highest-attribution k-mers were modestly over-represented within functional ORF2 coding regions rather than being distributed uniformly across the length of LINE-1. These findings establish TrAP as a transformer-assisted framework for improving LINE-1 quantification in short-read RNA-seq datasets.

## 2. Results

We evaluated TrAP as an integrated LINE-1 analysis pipeline through a sequential validation framework as follows: (1) characterization of the simulated training dataset; (2) interpretation of the trained classifier to establish which sequence features drive its predictions; (3) evaluation of downstream LINE-1 abundance estimation in synthetic datasets containing controlled LINE-1 insertions of varying size, mutation rate, deletion probability, and truncation; (4) benchmarking of short-read RNA-seq abundance estimates from HEK293 cells against long-read 5′RACE reference measurements; (5) and application of classifier-derived scoring framework to the 5′RACE dataset to assess whether model-derived LINE-1 scores reproduced broad chromosome-level patterns observed with alignment-based analysis.

### 2.1. Dataset and Model Diagnostics

To characterize the composition and structure of the training dataset, we quantified the number of reads assigned to each class (L1HS, L1PA, and NEGATIVE) across the training, validation, and test splits after aligning the simulated RNA-seq reads to the GRCh38.p14 genome assembly with GENCODE v48 splice junctions and partitioning them at the transcript level (Table 1). This dataset served as the foundation for training and evaluating the read-level classifier. Table 1 shows that LINE-1-positive (L1HS and L1PA) constituted most of the labeled corpus. This distribution resulted from transcriptome-wide read simulation followed by deliberate sampling. NEGATIVE reads were subsampled with lower coverage to produce approximately balanced LINE-1-positive and NEGATIVE classes for training.

Beyond raw sample size, we assessed sequence complexity and redundancy to guide k-mer selection for downstream modeling. Figure 1 shows that entropy increased with k-mer length and approached a plateau near *k* = 16, indicating diminishing gains in measured sequence-complexity within the transcript-derived training corpus. We did not interpret this plateau as evidence that longer k-mers are biologically uninformative in all settings. The plateau was considered together with sampling reliability, tokenizer behavior, encoded sequence length, the 1280-position model input budget, vocabulary constraints, and computational feasibility in selecting *k* = 17 as an operational representation for downstream ALBERT-based modeling. Figure 2 shows the marginal information gain contributed by each additional base of k-mer context, which falls to a negligible level beyond *k* ≈ 16 and identifies *k* = 17 as the shortest k-mer length at the onset of this plateau. We therefore selected *k* = 17 as the preferred operating setting for the current dataset, tokenizer, and ALBERT configuration, balancing local sequence specificity against vocabulary sparsity and encoded sequence length. This selection represented a study-specific design choice and should not be interpreted as a universal optimum for other genomic applications. To assess how far the plateau could be interpreted, entropy was additionally estimated with bias-corrected estimators (Miller–Madow and Chao-Shen, Figure 3), and a rarefaction analysis re-estimated entropy across corpus subsample fractions (Figure 4). The corrected estimators agreed with one another, and the rarefaction curves overlapped, up to *k* ≈ 14 beyond that length, the corrections diverged, and the curves separated by subsample fraction. The plateau therefore occurred at the edge of the range over which the available corpus could distinguish further entropy gains from sampling limitations.

We next evaluated how different tokenization strategies influenced sequence encoding and encoded length [29], and we adopted a canonical k-mer tokenizer for the reported pipeline. Following the same canonical-k-mer convention used by Salmon, this tokenizer mapped each overlapping stride-1 k-mer to exactly one token, so every k-mer in a read is represented and coverage is 100% by construction at all evaluated k. Because the mapping is one token per k-mer, the encoded sequence length was deterministic and equaled the number of overlapping k-mers in the read (read length − *k* + 1), which for a 150 bp read is 134 tokens at the operational *k* = 17. This lossless, fixed-length encoding avoided the vocabulary-coverage limits of WordLevel tokenization and the variable sub-word segmentation of learned tokenizers such as WordPiece and SentencePiece, while producing a compact representation suitable for ALBERT-based training.

We assessed the read-level classifier on the validation set using per-class and macro-averaged F1, precision, recall, the area under the precision-recall curve (PR-AUC), and the area under the receiver operating characteristic curve (ROC-AUC) (Table 2). The classifier achieved strong overall performance, with an accuracy of 0.959, a macro-averaged F1 of 0.865, and a Matthews correlation coefficient of 0.920. Per-class performance was high for the L1PA and NEGATIVE classes and lower but substantial for the rarer L1HS class, consistent with its sequence similarity to L1PA. Given that the validation split was also used for checkpoint selection and early stopping, we additionally evaluated the held-out test split described in Table 1. The classifier achieved an accuracy of 0.959 and a macro-averaged F1 of 0.860 on the test split, compared with 0.959 and 0.865, respectively, on the validation split; complete per-class metrics and the confusion matrix are reported in Table S1. We separately evaluated masked-language-model pretraining of the ALBERT encoder as an optional initialization step; because this pretraining did not improve downstream read-level classification, it was not used in the final pipeline.

Finally, we optimized classifier hyperparameters using Population-Based Training (PBT), which updates model weights and hyperparameters concurrently during training. The primary tuning objective was validation F1, with wall-clock runtime serving as a secondary criterion. Figure 5 shows the 16 trials with the shortest training time on an identical read sample. These trials emerged from a total of 128 trials configured to carry forward those with the highest validation F1 and exceeding 0.95 on evaluation accuracy, precision, and recall. Runtime was used to separate trials of comparable F1. Because PBT mutates hyperparameters online while cloning weights from high-performing runs, it efficiently converges on competitive solutions without exhaustive grid search.

### 2.2. Model Interpretation and Sequence Attribution

Attribution scores captured classifier behavior that raw attention did not convey. Across all LINE-1 k-mers, the last-layer head-averaged attention received by each k-mer was essentially uncorrelated with its integrated-gradients attribution (Spearman *ρ* = 0.01, Pearson *r* = −0.04; Figure 6). This confirms that raw attention is not a reliable importance proxy and motivates the gradient-based attribution used here.

Aligning the positive-class k-mers to the L1.3 consensus sequence mapped 97,418 k-mers to annotated LINE-1 domains. K-mers in the highest attribution decile were modestly over-represented in the ORF2 C-terminal domain (1.16-fold enrichment; 95% confidence interval 1.02–1.29). Overlapping stride-1 k-mers from the same read were not independent observations, the confidence interval was obtained by bootstrap resampling of reads rather than individual k-mers. A weaker, nonsignificant enrichment was observed in the ORF2 reverse transcriptase domain, whereas no enrichment was detected in ORF1 (Figure 7). Thus, the classifier assigned greater importance to k-mers mapping to ORF2 coding regions rather than distributing attribution uniformly across the LINE-1 retroelement. Of the eight consensus partitions examined, the ORF2 C-terminal region was the only one significantly enriched in classifier attribution; the 5′UTR was not enriched (0.68-fold, not significant). Although ORF0 was not included as a separate consensus partition, we performed an additional positional analysis of its footprint within the 5′UTR, corresponding to the 71-codon antisense open reading frame at consensus positions 235–450. The 853 k-mers mapping to this footprint were depleted of top-decile attribution relative to the full consensus (0.57-fold; odds ratio 0.55, 95% CI 0.40–0.73; *p* = 1.2 × 10^−5^) but did not differ significantly from the remainder of the 5′UTR (0.84-fold; *p* = 0.18). Because canonical k-mer tokenization collapses each k-mer with its reverse complement, this analysis identifies attribution by position within the 5′UTR but cannot specifically attribute the assignment to the antisense ORF0 reading frame. This attribution analysis was exploratory and complementary to the quantification benchmarks.

Figure 8 shows the model interpretations for four random paired-end reads in the dataset, labeled for LINE-1 presence or absence. The figure shows the relevance of scores assigned by the model to each k-mer in the sequences. Darker shades of green indicate higher importance, offering insight into the model’s decision-making process. The stronger attributions in the displayed NEGATIVE reads reflected sequence features supporting assignment to the NEGATIVE class, rather than features supporting LINE-1 recognition.

### 2.3. Evaluating Performance in a Synthetic Dataset

To enhance the biological fidelity and complexity of the evaluation framework, we examined synthetic LINE-1 insertions in the chromosome 1 transcript sequences of the GENCODE v48 annotation (GRCh38.p14). Chromosome 1, being the largest human chromosome, is particularly relevant due to its high gene density and the presence of critical oncogenes such as MCL1 and MYCL1. Restricting the simulation to one chromosome made the full parameter grid computationally feasible across all methods, although the results may not generalize across the genome. We inserted full-length LINE-1 elements at copy numbers increasing in powers of two, from 2^5^ (32) to 2^13^ (8192) copies per simulated dataset. To simulate natural sequence variability, each insertion was subjected to point mutations independently per position with a probability of 1% with the replacement base drawn uniformly from the four bases, giving an effective substitution rate of 0.0075. Internal deletions were also introduced to mimic reverse transcription errors, with deletion probabilities varying from 0% to 10% in 2.5% increments per insertion. Where a deletion occurred, its start and end were each drawn uniformly over the element, so expected deletions were long rather than short indels. Additionally, to replicate incomplete reverse transcription events, the 5′ end of each insertion was truncated at a position drawn uniformly within the first 15% of the element length, corresponding to the 5′ UTR fraction derived from L1.3, with all sequence 3′ of that position retained. Short duplicated sequences with lengths drawn uniformly from 5 to 15 bp and random composition were added to both flanks of the insertion site to simulate target-site duplications. The insertion strand was drawn at random, and the element was reverse-complemented for minus-strand insertions. All insertions were based on annotations from the L1base database [30], ensuring realistic representation of LINE-1 retroelements. Paired-end reads were simulated from the modified transcript sequences rather than from a modified chromosome. Coverage was uniform across transcripts and therefore did not reproduce the expression-weighted abundance distribution of a biological RNA-seq library. The simulation parameters included a read length of 150 base pairs, an average fragment size of 500 base pairs, and a standard deviation of 10 base pairs. This standard deviation was narrower than that of a typical sequencing library and was selected to limit variability unrelated to read attribution, the quantity evaluated in this benchmark. Sensitivity to wider fragment-length distributions was not assessed. The resulting reads were aligned to the reference genome using STAR with a consistent benchmarking alignment configuration so that all alignment-dependent comparator tools were evaluated from the same alignment files. This setup enabled rigorous performance evaluation of the method.

To estimate the abundance of LINE-1 elements, we employed a range of tools and pipelines, including HTSeq [31], TEtranscripts [32], Salmon [33], and L1EM [34], together with two pipelines coupling the ALBERT classifier to downstream abundance estimation: AlbertSalmon, which uses Salmon, and AlbertEM, which uses an expectation–maximization algorithm. Comparator methods were selected to represent complementary strategies for LINE-1 abundance estimation. HTSeq was included as a conventional feature-counting baseline, TEtranscripts as a TE-aware counting method, Salmon as a lightweight probabilistic quantifier, and L1EM as a LINE-1-focused expectation–maximization approach. ALBERT was coupled specifically to Salmon and to an EM-based estimator because these approaches are compatible with the intended role of the classifier as a read-level sequence-discrimination step upstream of probabilistic abundance estimation. HTSeq and TEtranscripts were retained as independent alignment-based baselines so that ALBERT filtering effects would not be conflated with their internal counting assumptions.

Table 3 summarizes the purpose, assumptions, and key features of comparator tools and provides context for their roles in abundance estimation.

The inserted elements were included in the references and annotations supplied to every quantification method and were therefore present in the search space of each method. For count-based abundance estimates, observed LINE-1 read counts were converted into an insertion-equivalent abundance estimate, the fragment contribution attributed to those elements, by normalizing against the number of reads expected from a LINE-1 feature of a given effective length under the simulated coverage model. Expected reads were calculated as:

$$\mathrm{Expected}\;\mathrm{Reads}=\frac{\mathrm{Effective}\;\mathrm{LINE}1\;\mathrm{Length}\times \mathrm{Coverage}}{\mathrm{Mean}\;\mathrm{Fragment}\;\mathrm{Length}}$$

The insertion-equivalent abundance was then estimated as:

$$\mathrm{Estimated}\;\mathrm{Abundance}=\frac{0.5\times \mathrm{Observed}\;\mathrm{Read}\;\mathrm{Count}}{\mathrm{Expected}\;\mathrm{Reads}}$$

where the mean fragment length was measured from each alignment file rather than taken from the nominal simulation setting, and the factor of 0.5 converted paired-end read counts to fragment counts. Thus, the quantity plotted in Figure 9 represents the summed estimated insertion-equivalent abundance across LINE-1 elements for each simulated insertion level, rather than a unitless observed/expected recovery ratio.

To evaluate the accuracy of abundance estimates, we generated two key visualizations. The first plot summarized the total estimated abundance across all inserted LINE-1 elements. Figure 9 shows the estimated abundance of LINE-1s across all deletion probabilities for each insertion level, with the red line denoting expected LINE-1 insertions. The *x*-axis represents the insertion level, where each level corresponds to a Chromosome 1 transcript set containing a specific number of randomly inserted LINE-1 elements. The *y*-axis shows the summed estimated insertion-equivalent abundance across LINE-1 elements for each simulated dataset. Because this quantity was aggregated across elements, a method could approximate the expected total despite allocating abundance inaccurately among individual elements, as observed for HTSeq. AlbertEM and L1EM underestimated aggregate abundance at lower insertion levels and overestimated it at higher levels. AlbertSalmon, Salmon, and HTSeq provided the closest aggregate estimates, whereas TEtranscripts generally underestimated abundance. This pattern has been reported as a limitation in transposable element quantification [35].

A scatter plot was then used to compare estimated abundance against expected values derived from known synthetic insertions, and the coefficient of determination (*R*^2^) was used to quantify the accuracy of each method. As shown in Figure 10, each panel displays abundance in the native output units of the corresponding method; therefore, absolute magnitudes were not directly comparable across panels, and method performance was compared using the within-method *R*^2^. AlbertSalmon achieved the highest agreement with the expected insertion levels, with a pooled *R*^2^ of 0.99 across the full synthetic grid, and therefore selected as the preferred TrAP implementation for downstream analyses. Its performance remained consistent across the design, including datasets containing highly mutated insertions. The stratified analysis showed that insertion load, rather than deletion probability, was the principal factor separating its performance from that of the other methods. Notably, the EM-based pipelines produced abundance estimates that consistently appeared above the regression line, and with abundance values significantly inflated relative to other methods. These patterns account for the overestimation of total insertions observed in Figure 9, indicating that the pipelines systematically overestimate abundance across all insertion levels.

To quantify variation across the synthetic design and compare the methods formally, we stratified the analysis by insertion level and deletion probability. Because the broad dynamic range across the full simulation grid can dominate the pooled *R*^2^, comparisons among methods were based primarily on per-stratum *R*^2^ values. Across the nine insertion-level strata, AlbertSalmon had the highest per-stratum *R*^2^ of all methods tested, with a mean of 0.947 ± 0.047, and a range of 0.865–0.991 (Table 4). It also had the smallest dispersion; the standard deviations of the other methods ranged from 0.116 to 0.249. AlbertSalmon ranked first against every comparator in all nine insertion-level strata (two-sided paired Wilcoxon signed-rank test, *p* = 0.0039) and all five deletion-probability strata (*p* = 0.0625). These are the minimum attainable two-sided *p* values at *n* = 9 and *n* = 5, respectively, and reflect complete directional consistency across strata rather than the magnitude of the differences. Paired bootstrap intervals over strata excluded zero for selected comparisons on both axes: AlbertSalmon versus Salmon +0.209 (95%CI, 0.122–0.319), and AlbertEM versus L1EM, +0.016 (95%CI, 0.005–0.024), across insertion-level strata, and +0.042 (95%CI, 0.041–0.043) and +0.011 (95%CI, 0.010–0.014), respectively, across deletion-probability strata. Because the resampling unit was a design stratum rather than an independent replicate, these intervals described consistency across the synthetic grid rather than inferential uncertainty across independent experiments. Deletion probability changed *R*^2^ by no more than 0.014 for any method over the range 0 to 0.1. By contrast, from 2^5^ to 2^13^ insertions, *R*^2^ declined by 0.114 for AlbertSalmon compared with 0.316 for AlbertEM, 0.347 for L1EM, 0.461 for Salmon, 0.619 for HTSeq, and 0.622 for TEtranscripts. The complete per-stratum values, paired tests, dispersion estimates, and bootstrap intervals are reported in Tables S2–S5.

### 2.4. Evaluating Performance Agreement with 5′RACE Long-Read Sequencing

To assess performance using real data, we first applied the RNA-seq pipeline to the Illumina-sequenced HEK293 dataset SRR3997504, containing 41,368,288 strand-specific paired-end reads (150 bp), with an average fragment length of 436 bp. The reads were aligned using STAR to provide a uniform alignment for the benchmarked tools that utilize BAM files for quantification, with multi-mapping information preserved consistently across the alignment-dependent methods used in this comparison. We estimated LINE-1 abundance using four methods: Salmon, L1EM, and AlbertSalmon or AlbertEM. To harmonize the short-read RNA-seq estimates with the 5′RACE reference, we restricted the comparison to RepeatMasker-annotated LINE-1 elements consistent with sense-oriented full-length or near full-length transcriptional potential, including elements extended downstream by 400 bp as recommended in the L1EM framework. Here, full-length refers to the genomic annotation status of the LINE-1 element used for filtering, not to full-body physical coverage by an individual 5′RACE read.

As an orthogonal reference, for promoter-initiated LINE-1 transcription, we utilized the PacBio 5′RACE dataset SRR4099955 from HEK293 cells [36]. This dataset was generated using a LINE-1-specific primer and yielded approximately 1237 bp cDNA products from the 5′ ends of LINE-1 transcripts rather than the full ∼6 kb length of canonical LINE-1 retroelements. Therefore, the 5′RACE reads measured locus-level concordance with 5′ transcription initiation, not total LINE-1 transcript abundance. Within this scope, the protocol enriched for RNAs transcribed from the LINE-1 promoter and provided the most quantitative view of LINE-1 expression available at locus-level resolution. Accordingly, this benchmark tested whether read-level classification assigned promoter-initiated signal to the correct loci, whereas validation of total abundance estimation relied on the synthetic benchmark, for which ground truth was known. The short-read and 5′RACE datasets derive from different sequencing projects, so this comparison relates independent HEK293 preparations. Following Deininger et al. [36], we quantified LINE-1 reads using RepeatMasker annotations and calculated their proportion relative to total reads. Most detected elements were from the L1HS and L1PA2 subfamilies, consistent with their known retrotransposition activity in human cells. Therefore, each point in Figure 11 represents an individual RepeatMasker-annotated LINE-1 element/locus retained after this filtering procedure. The downstream estimator distributes abundance across annotated LINE-1 loci but cannot distinguish signal originating from an annotated copy from an unannotated or de novo insertion carrying similar sequence. Because the benchmarked tools report abundance in different units, we retained each method’s native output scale and interpreted cross-method comparisons as concordance in relative LINE-1 abundance rather than as direct equivalence of physical units. Salmon-based methods report abundance as TPM, whereas EM-based methods return estimated read assignments that were normalized as RPM. TPM includes feature-length normalization before scaling, whereas RPM normalizes assigned reads by sequencing depth only. RPM was calculated as *RPM_i_* = (10^6^ × *r_i_*)/*N*_mapped_, where *r_i_* is the estimated number of reads assigned to LINE-1 element *i* and *N*_mapped_ is the total number of mapped reads considered in the analysis.

The AlbertSalmon pipeline achieved the highest correlation with the 5′RACE data (*R*^2^ = 0.91), followed by Salmon (*R*^2^ = 0.86), the EM pipeline (*R*^2^ = 0.85), and L1EM (*R*^2^ = 0.83). Because the methods report different abundance units, these *R*^2^ values are interpreted here as measures of relative concordance with the 5′RACE reference across LINE-1 elements rather than as evidence that TPM- and RPM-scaled estimates are numerically interchangeable. The distribution of LINE-1 abundance values in the HEK293 5′RACE reference was highly right-skewed, with one dominant L1HS locus, a smaller number of moderately expressed L1HS/L1PA2 elements, and a long tail of low-abundance loci. Therefore, most points in Figure 11 were concentrated near the lower-left region of the scatter plots. This pattern likely reflects the underlying 5′RACE reference dataset rather than a plotting artifact. The circled point in Figure 11 marks the L1PA2 locus with the largest residual from the fitted regression. However, leave-one-out analysis identified the dominant L1HS locus, rather than the circled locus, as the influential observation, demonstrating that residual magnitude and regression leverage are distinct quantities.

The robustness of this comparison was then assessed using leave-one-out analyses and rank-based correlation. We defined a pre-specified matched set of 156 loci with finite estimates from all four methods and non-zero 5′RACE signal. Within the matched set, the paired quantifier ranking was unchanged after excluding the dominant L1HS locus (chr22:28663283-28669315), the largest-residual L1PA2 locus (chr1:174843226-174849243), or both. Excluding the dominant locus reduced *R*^2^ for every method by 0.22–0.27, confirming its strong leverage, whereas excluding the largest-residual locus increased *R*^2^ for every method without changing the ranking (Table 5). The composition and dynamic range of the reference constrained what this comparison could resolve. Of 1483 annotated L1HS/L1PA2 loci, 998 had nonzero 5′RACE signal, and only nine contributed more than 1% of the total. Across these nonzero loci, the minimum, first quartile, median, third quartile, 90th percentile, 99th percentile, and maximum contributions to the total 5′RACE signal were 0.0031%, 0.0031%, 0.0125%, 0.0343%, 0.112%, 0.901%, and 12.21%, respectively. By subfamily, 295 of 400 L1HS loci and 703 of 1083 L1PA2 loci had nonzero signal, with maximum contributions of 12.21% and 3.63%, respectively. Given this right-skewed distribution, we reported rank-based correlation alongside Pearson *R*^2^. Across the matched loci, Spearman *ρ* was 0.449 for AlbertSalmon, 0.425 for Salmon, 0.459 for AlbertEM, and 0.448 for L1EM, preserving both paired within-quantifier comparisons.

Restricting the comparison to loci overlapping a full-length L1Base2 element with a body longer than 6 kb left concordance essentially unchanged. Among the 136 matched loci retained, *R*^2^ was 0.869, 0.797, 0.877, and 0.845 for AlbertSalmon, Salmon, AlbertEM, and L1EM, respectively, compared with 0.863, 0.783, 0.873, and 0.841 in the All-matched loci set. Both paired margins were preserved (Δ*R*^2^ of +0.072 and +0.032; Table 5). The locus-level result was therefore robust in restricting the evaluation targets to L1Base2-curated full-length elements. The locus-level result is therefore not an artifact of scoring RepeatMasker-defined intervals.

As a sensitivity analysis, we multiplied the L1HS estimates in the matched set by the aggregate precision-to-recall ratio of 1.159. This increased *R*^2^ from 0.863 to 0.881 for AlbertSalmon and from 0.873 to 0.893 for AlbertEM, widening the corresponding paired margins from +0.080 to +0.098 and from +0.033 to +0.052 (Tables S6 and S7). Thus, the observed L1HS classification deficit did not account for the improvement in locus-level concordance. Because this global adjustment assumed uniform classification performance across loci, it was used only as a sensitivity analysis.

### 2.5. Performance Evaluation of 5′RACE Long-Read Sequencing in HEK293 Cells

To further explore whether classifier-derived signals reflected broad chromosomal LINE-1 activity patterns, we analyzed the 5′RACE long-read dataset (PacBio, SRR4099955) from HEK293 cells and aggregated classifier-assigned read counts by chromosome, assigning each read to a single class by one-hot argmax across the three class posterior probabilities. Because the classifier was trained on short paired-end read representations, PacBio 5′RACE reads were not interpreted as intact full-length ALBERT inputs. Instead, each long-read was fragmented in silico into simulated paired-end short reads, which were then k-mer tokenized and classified, and the resulting per-class read counts were summed by chromosome for exploratory comparison with the alignment-derived reference. As shown in Figure 12 these chromosome-level counts were compared with the per-read LINE-1 overlap fraction obtained from alignment with the Burrows-Wheeler Aligner (BWA, v0.7.18), summed per chromosome [37]. Because chromosome-level aggregation can be influenced by chromosome size, LINE-1 density, and other broad genomic features, this analysis should be interpreted as an exploratory concordance assessment rather than a locus-specific validation of abundance estimation. Within that scope, the two approaches captured broadly similar chromosome-level patterns for the most transcriptionally active LINE-1 classes in this dataset.

To compare the consistency of the prediction against traditional alignment-based methods, we computed the Pearson correlation between the chromosome-level one-hot argmax read count for each class and the per-read LINE-1 overlap fraction from the BWA alignment, summed per chromosome, across chromosomes in the 5′RACE dataset. As in Table 5, the alignment axis was restricted to curated full-length elements: RepeatMasker LINE-1 loci were first intersected with the union of the full-length intact and full-length non-intact L1Base2 tracks, and the retained intervals were then required to be at least 6 kb long. Reads overlapping only loci removed by this restriction are counted in the alignment NEGATIVE category. As shown in Figure 13 the strongest agreement was observed for L1HS (*r* = 0.96), whereas L1PA2 showed moderate concordance (*r* = 0.83). We therefore interpret Figure 13 as supporting class-dependent chromosomal concordance between classifier-derived scores and alignment-based counts, rather than uniform equivalence across all output classes. The NEGATIVE class is a classifier rejection category and was not interpreted as a LINE-1 abundance measure. Using the one-hot argmax assignment applied during evaluation, 38.1% of reads from annotated L1HS elements and 46.3% of reads from annotated L1PA elements were assigned to NEGATIVE. Consequently, NEGATIVE-class counts may covary with LINE-1 abundance because this category includes a substantial fraction of genuine LINE-1 reads. The correlation between NEGATIVE-class counts and the L1PA2 alignment signal (*r* = 0.74) persisted after adjustment for either the total per-chromosome LINE-1 alignment signal (partial *r* = 0.63, *p* = 0.0011) or the number of annotated LINE-1 loci per chromosome (partial *r* = 0.65, *p* = 0.0005). Coefficients for every class pairing, under these covariates as well as under chromosome length, are reported in Tables S8 and S9. These findings do not support interpreting NEGATIVE as a clean control or attributing its correlation solely to broad compositional differences among chromosomes. Reads assigned to NEGATIVE are discarded before quantification, making these assignments a source of reduced LINE-1 recall. Consistent with the influence of the dominant chromosome 22 L1HS locus, leave-one-chromosome-out analysis identified chromosome 22 as the most influential chromosome. Removal of chromosome 22 reduced the L1HS correlation from 0.96 to 0.84 but did not eliminate the positive association. The complete leave-one-chromosome-out results are reported in Table S10.

## 3. Discussion

The biological questions motivating the measurement of LINE-1 transcript abundance, including whether LINE-1 RNA functions as a regulator or reflects a cellular state, can be addressed only to the extent that the measurement distinguishes transcripts derived from transcriptionally active elements from incidental transcription through LINE-1 sequences. Across the matched and full filtered analyses, AlbertSalmon exceeded Salmon and AlbertEM exceeded L1EM. Rank-based correlation further preserved both comparisons on the matched locus set. Consequently, the paired within-quantifier direction was preserved across every sensitivity analysis examined.

Among 1483 annotated L1HS/L1PA2 loci, 998 had detectable 5′RACE signal, but only nine contributed more than 1% of the total, and the absolute *R*^2^ for every method was sensitive to the leverage of a single dominant L1HS locus. Nevertheless, excluding the dominant locus, the largest-residual L1PA2 locus did not change either of the paired within-quantifier rankings. The locus-level claim is therefore based on the stable paired differences rather than the leverage-sensitive absolute *R*^2^ values.

The enrichment of high-attribution k-mers within the ORF2 coding region permits a biological interpretation, although the analysis was not designed to test the basis of that enrichment and the following reading is therefore post hoc. Two features of LINE-1 sequence organization are consistent with it. First, because most genomic LINE-1 copies are 5′-truncated, sequences toward the 3′ portion of ORF2 are retained across a larger fraction of the genomic copy population and may therefore supply a greater share of the sequence information available to a read-level classifier [38]. Second, the ORF2 carboxy-terminal region is subject to functional constraint: its cysteine-rich domain is required for retrotransposition [39] and has been resolved in recent structural characterizations of the ORF2-encoded protein (ORF2p) [40]. The classifier may therefore have weighted sequences that are both widely retained among genomic copies and constrained by ORF2p function rather than an arbitrary region of the element. Attribution, however, identifies features that are informative for the classification task; it does not establish that the corresponding k-mers are themselves functionally important, and distinguishing these possibilities will require targeted experimental or computational perturbation analyses rather than reanalysis of the present data.

While TrAP offers valuable insights into LINE-1 sequence abundance, several limitations must be acknowledged. First, TrAP does not determine the transcript form or promoter of origin of the signal, and subfamily-level estimates can include substantial contributions from copies that retain LINE-1 sequence but do not produce autonomous LINE-1 transcripts [41]. Transcript-form-agnostic classification may capture biologically relevant LINE-1-derived signal, but cannot establish whether that signal originated from autonomous, chimeric, internally initiated, or passive transcription. This limitation most directly constrains regulatory interpretation because subfamily-level sequence content alone cannot distinguish passive intronic co-transcription from other transcript forms. Abundance attributed to the aggregate L1PA class has limited biological specificity because the class groups transcriptionally competent young subfamilies with older primate subfamilies that are not transcriptionally competent. Second, the read-level labels were derived from RepeatMasker annotations, which may bias the classifier toward annotated LINE-1 sequence features that do not by themselves establish discovery of entirely annotation-independent LINE-1 biology, and evidence of biological performance therefore rests on the downstream benchmarks instead. Restricting the 5′RACE comparison to L1Base2-curated full-length elements accordingly established independence in the selection of evaluation loci, but not in the training labels or underlying interval annotations. Third, transformer-based inference also carries computational overhead relative to lightweight alignment or pseudo-alignment tools, so any claim about efficiency or scalability would require dedicated benchmarking of wall-clock runtime, memory use, and throughput. Fourth, transcript-level partitioning prevented reads from the same transcript from entering different splits but did not control sequence similarity across splits, particularly among isoforms sharing exons and closely related LINE-1 copies. Accordingly, because gene-level or sequence-similarity-aware partitioning was not evaluated, the reported classification metrics may represent an upper-bound estimate of generalization to unrelated sequences. The contribution therefore lies in integrating read-level classification with downstream abundance estimation, with classification consistently improving benchmark concordance without changing the underlying quantifier.

Transcript abundance should not be interpreted as a direct measure of ORF2p production, retrotransposition competence, or retrotransposition activity. Whether a transcript-level LINE-1 readout carries regulatory, pharmacodynamic, or diagnostic information remains to be determined in appropriately designed biological and clinical studies. For example, an assay intended for pharmacodynamic assessment would need to distinguish suppression of transcriptionally active elements from changes in passive or background LINE-1-associated signal [42]. Such a readout would also need to resolve locus-level heterogeneity because LINE-1 transcript abundance in non-small-cell lung cancer, for example, varies with the genomic context of individual elements rather than uniformly across the family [4].

Three directions follow most directly from these results. The k-mer length was selected based on information-theoretic and tokenization-efficiency criteria rather than a downstream ablation; whether single-nucleotide or longer-k-mer tokenization improves read classification or abundance estimation under matched model capacity and training conditions therefore remains untested. Such an ablation would require rebuilding the tokenizer and retraining the classifier at each k-mer length, which was beyond the scope of the present study. The benchmarks comprised one synthetic design and one cell line, so validation in further biological datasets is needed to establish the generalizability of the locus-level findings. Because TrAP classifies LINE-1-derived reads directly from sequence content without relying exclusively on alignment, a promising future direction is its extension to single-cell RNA-seq (scRNA-seq) data, where quantifying transposable-element expression at the single-cell level remains a major challenge and for which an alignment-free transformer approach may be particularly well suited. A systematic comparison of alternative classification stages, from simpler k-mer models to larger architectures, will be a future priority.

Although the read-level formulation of TrAP could potentially be extended to other repetitive sequences and poorly characterized genomic annotations, its performance beyond LINE-1 has not been evaluated. The classifier is also orientation-agnostic; a read spanning a 5′-inverted LINE-1 junction is classified once as LINE-1-positive rather than resolved as an inversion event. Canonical k-mer tokenization maps each k-mer and its reverse complement to the same token, thereby discarding strand information at the representation level. TrAP therefore does not distinguish sense from antisense LINE-1 transcription, even in stranded data such as the HEK293 library. Moreover, its outputs describe read-level composition rather than insertion loci, which require integration with downstream frameworks. TrAP therefore contributes a novel integration of read-level sequence classification with established abundance estimation, complementing alignment-based approaches when multi-mapping and repetitive-sequence ambiguity limit quantification.

## 4. Materials and Methods

Our workflow initially generated labeled training reads from GENCODE v48 transcripts (GRCh38.p14) [43] and proceeded through k-mer-length evaluation, tokenization, and model training. Here, tokenization denotes conversion of each overlapping nucleotide k-mer into a unique integer identifier from a fixed vocabulary. The primary artifact of record was the per-read label, which classified each read pair as “L1HS”, “L1PA”, or “NEGATIVE” (all other sequences), and provided supervised targets for the classification model. We formulated LINE-1 read detection as a supervised multi-class sequence-classification problem. The trained classifier was integrated into two abundance-estimation pipelines: (i) AlbertEM, which couples the classifier to an expectation–maximization (EM) estimator, and (ii) AlbertSalmon, which couples it to Salmon. Salmon is a probabilistic transcript quantifier, while EM probabilistically assigns ambiguous reads among annotated LINE-1 elements. Reads predicted as NEGATIVE were filtered before abundance estimation using a probability threshold of *τ* = 0.5. The threshold was selected as a permissive approximation of three-class argmax classification rather than as an independently tuned parameter. Under this criterion, reads with *P*(NEGATIVE) ≥ 0.5 are necessarily assigned to NEGATIVE by the argmax rule because the three class probabilities sum to one. The pipelines report abundance for LINE-1 elements defined by RepeatMasker annotations on GRCh38.p14 (NCBI RefSeq GCF_000001405.40). Salmon-based estimates were reported as transcripts per million (TPM), whereas EM-based read assignments were normalized as reads per million mapped reads (RPM). TrAP provided read-level classification and relied on downstream estimators for locus-level abundance. Figure 14 summarizes the workflow from sequence input through tokenization, classification, and abundance estimation.

### 4.1. Dataset Generation

This procedure generated a synthetic RNA-seq dataset to train the read-level classifier [44]. Synthetic short-read RNA-seq data were generated with the ART (Artificial Read Simulator, v2.5.8) tool [45] from the GENCODE v48 (GRCh38.p14) reference, configured to simulate paired-end Illumina reads with a read length of 150 bp. For the paired-end configuration, the mean fragment length was set to 500 bp and the fragment-length standard deviation was set to 10 bp. ART was run at a target coverage of 5 × against the GENCODE transcript input rather than expression-weighted RNA-seq abundance. This controlled simulation strategy was used to generate a reproducible training set with broad representation of transcript-derived sequence content for supervised read-level classification [44]. Synthetic reads were aligned to the GRCh38.p14 genome assembly using the Spliced Transcripts Alignment to a Reference (STAR) aligner with GENCODE v48 splice junction annotations [46]. STAR was configured to retain highly repetitive reads by allowing permissive multi-mapping, using --outFilterMultimapNmax 100 and --winAnchorMultimapNmax 100 [47]. These settings allowed reads with up to 100 reported mapping loci and up to 100 anchor multi-mapping loci to be retained for downstream annotation-based labeling. This configuration was intended to maximize sensitivity for LINE-1-derived reads while avoiding the incorrect interpretation of these alignments as supplementary or split-read alignments. Read labels were then assigned by intersecting alignments with RepeatMasker annotations of LINE-1 elements [48]. Read pairs were labeled “L1HS” or “L1PA” when at least half of either mate overlapped an annotated L1HS or L1PA locu, respectively; pairs were labeled “NEGATIVE” when neither mate overlapped an annotated LINE-1 locus. Pairs whose only overlap covered less than half of a mate were discarded. When a pair met the overlap criterion for multiple classes, one label was assigned according to the fixed priority L1HS > L1PA > NEGATIVE. The L1PA class comprised 38 RepeatMasker subfamilies matched by the L1P* prefix, spanning L1PA2 through the older primate subfamilies; the ancient, mammalian-wide L1M and L1ME subfamilies were not assigned to either LINE-1-positive class.

### 4.2. Optimal k-mer Length Determination

The k-mer pipeline processed DNA sequences into k-mer profiles by segmenting them into overlapping k-mers to generate fixed-size tokens, as summarized in the k-mer tokenizer step of Figure 14. The k-mers captured local sequence information and served as core analytical units, with individual profiles used to analyze sequence composition, regional conservation, and genetic variation. To guide this tradeoff, we quantified sequence complexity across k-mer lengths and treated k-mer length selection as an operational design choice for transformer tokenization rather than as a universal optimum. Increasing k can improve local sequence specificity, but it also increased the number of possible k-mers, reduced repeated observations of individual tokens, increased sparsity, and can affect tokenizer efficiency and model vocabulary usage. For each value of k, transcript sequences were decomposed into overlapping k-mers using a one-nucleotide sliding window. The empirical probability of each k-mer w was defined as:

$$p_{k}(w)=\frac{c_{k}(w)}{\sum_{w^{\prime }\in V_{k}}c_{k}(w^{\prime })}$$

where *c_k_*(*w*) is the observed count of k-mer *w* across the full transcript set and *V_k_* is the set of all observed k-mers of length *k*. Shannon entropy was then calculated for each k-mer length as:

$$H(P_{k})=-\sum_{w\in V_{k}}p_{k}(w)\log_{2}p_{k}(w)$$

where *P_k_* denotes the empirical k-mer frequency distribution at length *k*. These measures were used to identify the point at which additional increases in k produced diminishing gains in measured sequence-complexity signal. Entropy and redundancy analyses indicated an approximate plateau around *k* = 16. We therefore interpreted *k* = 16 as the information-theoretic plateau point and evaluated *k* = 17 as a conservative operational tokenization length immediately above this plateau. This choice was intended to preserve local sequence specificity while limiting sparsity, vocabulary burden, and encoded sequence length for ALBERT-based modeling. Because plug-in Shannon entropy is downward-biased when the number of possible k-mers is large relative to the sample, entropy was additionally estimated using the Miller–Madow and Chao-Shen bias corrections, and a rarefaction (subsampling) analysis was used to establish the range of k over which entropy estimates were sampling-stable.

### 4.3. Tokenization of GENCODE Transcript

Tokenization, a critical preprocessing step for applying ML to RNA sequences, was performed upon completion of the k-mer analysis. Sequence tokenization was performed using a deterministic canonical k-mer tokenizer with semantics matching those used by Salmon and Jellyfish, a fast, memory-efficient algorithm for counting k-mers [49]. Both tools rely on canonical k-mer handling to enable ultra-fast, alignment-free genomic analysis, and adopting the same k-mer semantics kept the tokenizer consistent with these established conventions. Each k-mer and its reverse complement were mapped to a single canonical identity, defined as the smaller of the forward and reverse-complement encodings, so that a k-mer and its reverse complement share one token. This scheme assigned exactly one token per k-mer, left no valid ACGT k-mer represented by an unknown token, and yielded a predictable encoded length in which a paired-end read mapped to approximately 271 tokens, consistent with the model position budget. Because tokens are derived directly from canonical k-mers rather than from a learned sub-word vocabulary, the vocabulary size was decoupled from the full space of possible k-mers, and the encoding was fully deterministic and reproducible. This canonical k-mer representation was also consistent with the downstream Salmon-based abundance-estimation step, which relies on the same canonical k-mer semantics, allowing the read-level classifier and the abundance estimator to share a common sequence-discretization scheme.

The vocabulary contained 65,541 tokens: 65,536 feature-hash buckets and five special tokens. Each canonical 17-mer was mapped deterministically to one of the hash buckets. Thus, every valid ACGT 17-mer, including novel k-mers generated by sequencing errors or sequence variants, received a valid token rather than an unknown token. This mapping necessarily permitted hash collisions. Non-ACGT characters, including N, were removed before k-merization; consequently, k-mers could span the position of a removed base. We additionally evaluated a content-preserving sub-word tokenizer (SentencePiece) and found that it did not improve read-level classification relative to the canonical k-mer tokenizer, consistent with the interpretation that assigning one token to each specific k-mer preserves the per-token sequence specificity needed to discriminate closely related LINE-1 subfamilies.

### 4.4. Selection of the Architecture

ALBERT was adapted for genomic sequence modeling by treating nucleotide-derived k-mer strings as the input language. Transcript-derived sequences and paired-end reads were first converted into overlapping k-mers and then encoded using a custom tokenizer implemented with PreTrainedTokenizerFast. The tokenizer inserted standard transformer special tokens using template processing, with single sequences formatted as [CLS] read [SEP] and paired inputs formatted as [CLS] Read Pair 1 [SEP] Read Pair 2 [SEP]. Because each read is encoded as one token per overlapping k-mer, reads shorter than 150 bp (e.g., after quality or adapter trimming) simply produced fewer tokens, and variable-length inputs were accommodated by padding each batch to a common length with [PAD] tokens together with an attention mask that excluded padded positions from the model output; reads exceeding the model position budget were right-truncated. ALBERT, a lightweight transformer-based model designed as an efficient alternative to BERT, was used for read-level sequence classification [50]. ALBERT enabled us to reduce model parameters while maintaining or improving performance. ALBERT reduces its parameter count using factorized embedding parameterization, which divides the embedding matrix into two smaller matrices. For read-level LINE-1 classification, the ALBERT encoder was coupled to a sequence-classification head using AlbertForSequenceClassification. The classifier consumed the tokenized read sequence, or paired-read representation, and generated a sequence-level embedding from the transformer encoder. This embedding was passed to a linear classification layer trained with cross-entropy loss to predict one of three labels: L1HS, L1PA, or NEGATIVE.

### 4.5. Training and Performance

We implemented the read-level classifier using the Hugging Face Transformers library (v5.9.0) with a PyTorch (v2.10.0) backend. The ALBERT classifier was trained directly on the supervised classification task from a random initialization, without a separate masked-language-model pre-training stage. To reduce redundancy-driven leakage between training and evaluation, the corpus was partitioned at the transcript level, so that all reads derived from a given transcript were assigned to a single split. Model parameters were updated by minimizing the cross-entropy classification loss with Adam optimization, and model quality was monitored using standard read-level classification metrics. Class imbalance was addressed during training with inverse-frequency balanced weighted cross-entropy, with the weight for class *c* was defined as *w_c_* = *N*/(*k* ⋅ *n_c_*). Based on the training-set frequencies of 60,639 L1HS, 2,439,877 L1PA, and 2,161,900 negative reads, the corresponding weights were 25.6294, 0.6370, and 0.7189 respectively. Their frequency-weighted mean was 1.0000, preserving the overall loss scale while increasing the relative contribution of the minority L1HS class. The class frequencies and weights are reported in Tables S11 and S12.

For paired-end read classification, read 1 and read 2 were encoded as paired inputs rather than concatenated without delimitation, using the format [CLS] Read Pair 1 [SEP] Read Pair 2 [SEP]. This formatting allowed the model to distinguish the two read components while preserving paired-end sequence context. Generalizability was evaluated primarily through downstream benchmarks: synthetic LINE-1 insertion experiments and HEK293 short-read RNA-seq comparisons against 5′RACE long-read reference measurements. We optimized hyperparameters for the read-level classifier with Ray Tune using the PBT scheduler. We selected PBT because it enables simultaneous optimization of model weights and hyperparameters during training and has been reported to improve convergence efficiency relative to conventional sequential hyperparameter-search strategies. The search was run for 128 trials with one-epoch perturbation intervals and selected on validation F1, over learning rate (log-uniform, 1 × 10^−5^ to 1 × 10^−3^), weight decay (uniform, 0 to 0.3), warmup ratio (0, 0.05, 0.1, or 0.2), and per-device batch size (8 or 16). The final selected classification configuration used a per-device training batch size of 16 with gradient accumulation of 2, giving an effective batch size of 64 across the two GPUs, a per-device evaluation batch size of 16, a learning rate of 5.0 × 10^−5^, a weight decay of 0.1796, and a warmup ratio of 0.1. Training ran for a maximum of 12 epochs with early stopping on validation F1 (patience 2); 9 epochs completed, requiring 18.4 h of wall-clock time on two NVIDIA A100 40 GB GPUs over 4,662,416 training read records.

### 4.6. Benchmarking, Abundance Estimation, and Comparator Settings

The benchmark alignment, which produced every BAM file supplied to the alignment-dependent comparators, retained up to 1000 reported loci per read and flagged all best-scoring alignments as primary (--outFilterMultimapNmax 1000 --outSAMprimaryFlag AllBestScore). Each comparator was then run at its own recommended multi-mapping setting for repetitive elements. HTSeq was run as htseq-count -f bam -r pos -s no -t exon -i gene_id --nonunique all, so that a read with several reported alignments is counted toward every annotated element it aligns to rather than discarded. TEtranscripts was run as TEcount --sortByPos --mode multi, which distributes multi-mapping reads across TE annotations by expectation maximization. L1EM was run with default parameters unmodified (realignNM 3, L1EM_NM 3, NMdiff 2, bwa_i 20, error probability 0.01, maximum start-to-start length 500, EM threshold 1 × 10^−7^), with the 400 bp flanking extension. Quantification used Salmon 2.1.2, htseq-count 2.1.2, TEcount 2.2.4 (TEtranscripts), and L1EM at upstream commit a50a237, and alignment and interval operations used STAR 2.7.11b, SAMtools 1.21, and BEDTools 2.31.1. Salmon was run on the synthetic and HEK293 benchmark datasets using a 31-mer decoy-aware index comprising the full transcriptome, the LINE-1 loci, and decoy sequences.

For the L1Base2-restricted analysis of the HEK293 comparison, loci were additionally required to overlap a full-length element in the L1Base2 Ens84.38 annotation [30]. Elements were required to have bodies longer than 6 kb to enrich for structurally complete elements capable of promoter-initiated transcription. This criterion retained 8554 L1Base2 elements. Of the 1483 annotated loci in the HEK293 comparison, 1259 overlapped a retained element, and 885 of these had nonzero 5′RACE signal. Requiring finite estimates from all four methods yielded the final matched set of 136 loci. For the chromosome-level comparison, the PacBio 5′RACE reads were not classified intact. Each long-read was first fragmented in silico into simulated 150 bp paired-end reads at 5× coverage, using a mean fragment length of 500 bp and a standard deviation of 30 bp. The resulting read pairs were tokenized and classified using the same procedure as for the short-read input.

Paired comparisons of locus-level concordance were performed on a pre-specified matched set of 156 loci with finite estimates from all four methods and non-zero 5′RACE signal, ensuring that every method was evaluated on identical loci. Loci with zero 5′RACE signal were excluded because zero represents non-detection rather than a measured abundance of zero. The aggregate effect of L1HS classification performance on abundance estimates was approximated using the ratio of precision to recall. If *T* is the true number of L1HS reads and *A* is the number assigned to L1HS, then *T*/*A* = precision/recall. The observed values therefore yielded an adjustment factor of 0.723/0.624 = 1.159 (Table S12). This factor was applied only to L1HS loci quantified after classification and was evaluated as a sensitivity analysis rather than an abundance correction, because aggregate classification metrics do not establish that misclassification is uniform across loci.

### 4.7. Transformer Model Interpretation

Model interpretation was performed using transformers-interpret (v0.10.0), an explainability package designed for Hugging Face transformer models. Because TrAP represents nucleotide sequences as k-mer tokens, we used a custom k-mer adaptation of SequenceClassificationExplainer to compute token-level attribution scores for the sequence-classification model. Positive attribution values indicate that a k-mer contributed toward the predicted LINE-1 class, whereas negative attribution values indicate that a k-mer contributed against that prediction. These attribution scores should therefore be interpreted as model-derived token attributions rather than raw attention weights. We computed token-level attributions with Layer Integrated Gradients over the ALBERT word-embedding layer (50 integration steps, [PAD] baseline) for 900 reads balanced across the L1HS, L1PA, and NEGATIVE classes (300 each). To confirm that these attributions capture classifier behavior beyond what raw attention conveys, we compared the last-layer head-averaged attention received by each k-mer with its integrated-gradients attribution across all LINE-1 k-mers. To assess whether high-attribution k-mers correspond to biologically meaningful LINE-1 features, we aligned the positive-class k-mers to the L1.3 consensus (GenBank L19088.1) and mapped them onto annotated LINE-1 domains. The background for each domain was the observed distribution of aligned k-mers across the annotated consensus regions, thereby accounting for the uneven representation of LINE-1 domains resulting partly from 5′ truncation. Enrichment of the highest attribution decile within each domain was quantified as the ratio of its observed fraction to the corresponding background fraction. A 95% confidence interval was obtained by bootstrap resampling of reads rather than individual k-mers because k-mer attributions within the same read were dependent.

## Figures and Tables

**Figure 1:**
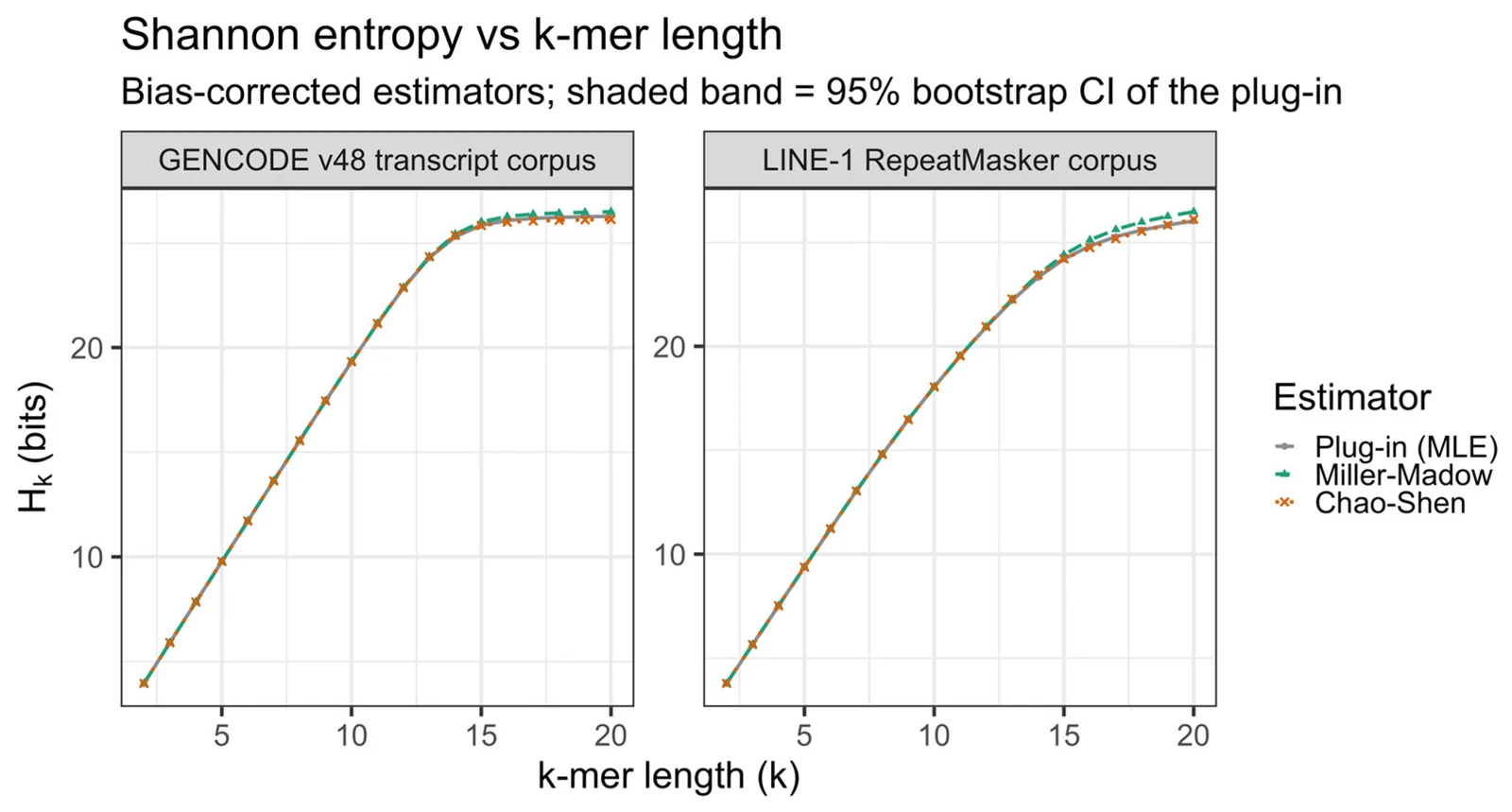
Information content of the transcript and LINE-1 k-mer spectra as a function of k-mer length. Shannon entropy is shown for each k, estimated with the plug-in (maximum-likelihood) estimator and with the Miller–Madow and Chao-Shen bias corrections; The 95% bootstrap confidence interval for the plug-in estimate is narrower than the plotted line (<0.001 bits). Entropy increases with k and approaches a plateau near *k* = 16. The plateau did not by itself distinguish information saturation from finite-sample limitations.

**Figure 2:**
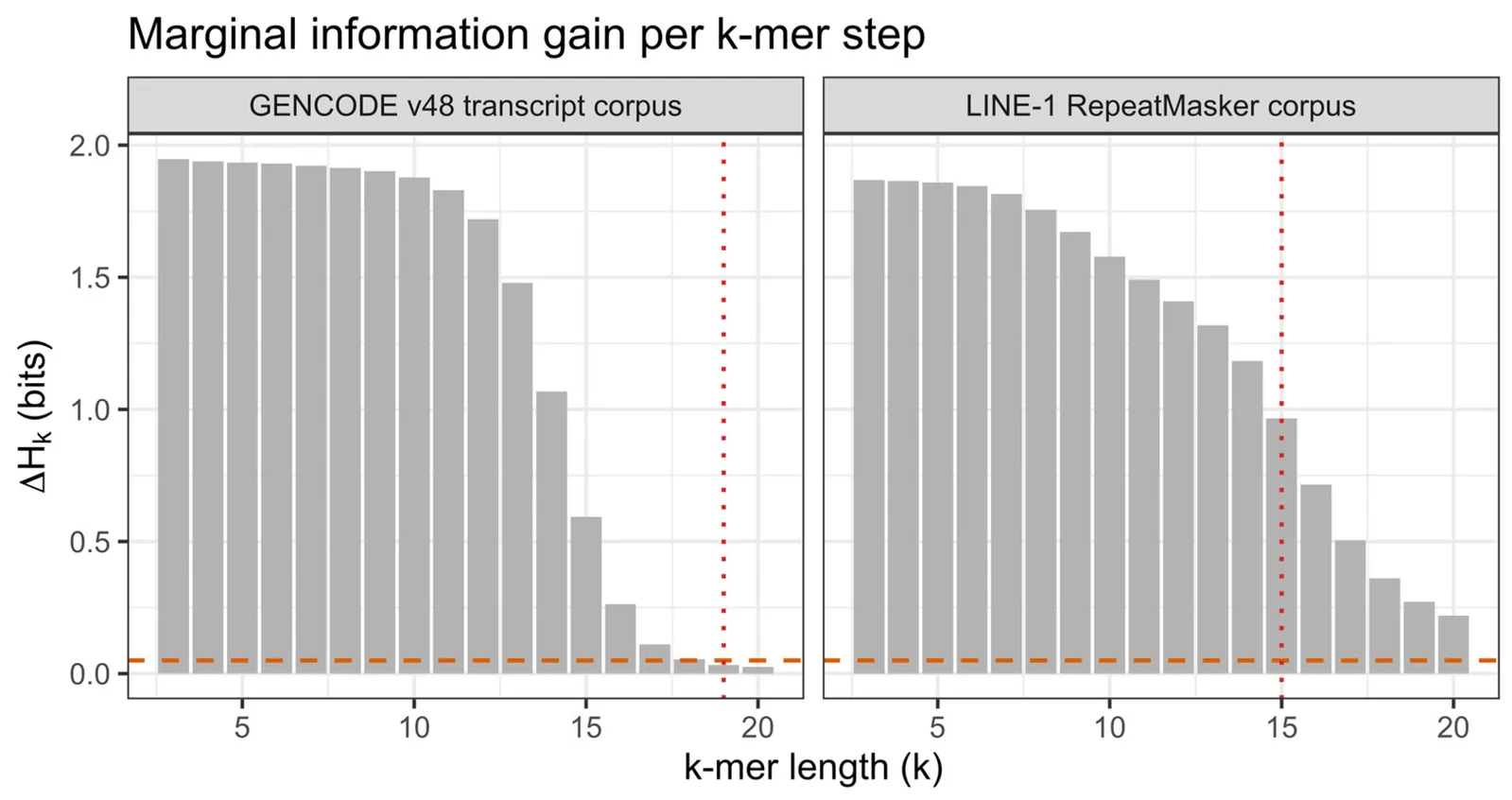
Marginal information gain per additional base of k-mer context. For each k, the change in bias-corrected entropy relative to *k* − 1 is shown; the gain diminishes progressively and falls to a negligible level beyond *k* ≈ 16. The horizontal dashed line indicates the 0.05 bits gain threshold, and the vertical dotted lines indicate the corpus-specific selection points (*k* = 19 for the GENCODE v48 transcript corpus and *k* = 15 for the LINE-1 RepeatMasker corpus). The recommended operating length, *k* = 17, is the shortest k-mer at the onset of this plateau, preserving local sequence specificity while limiting vocabulary sparsity and encoded-sequence length.

**Figure 3:**
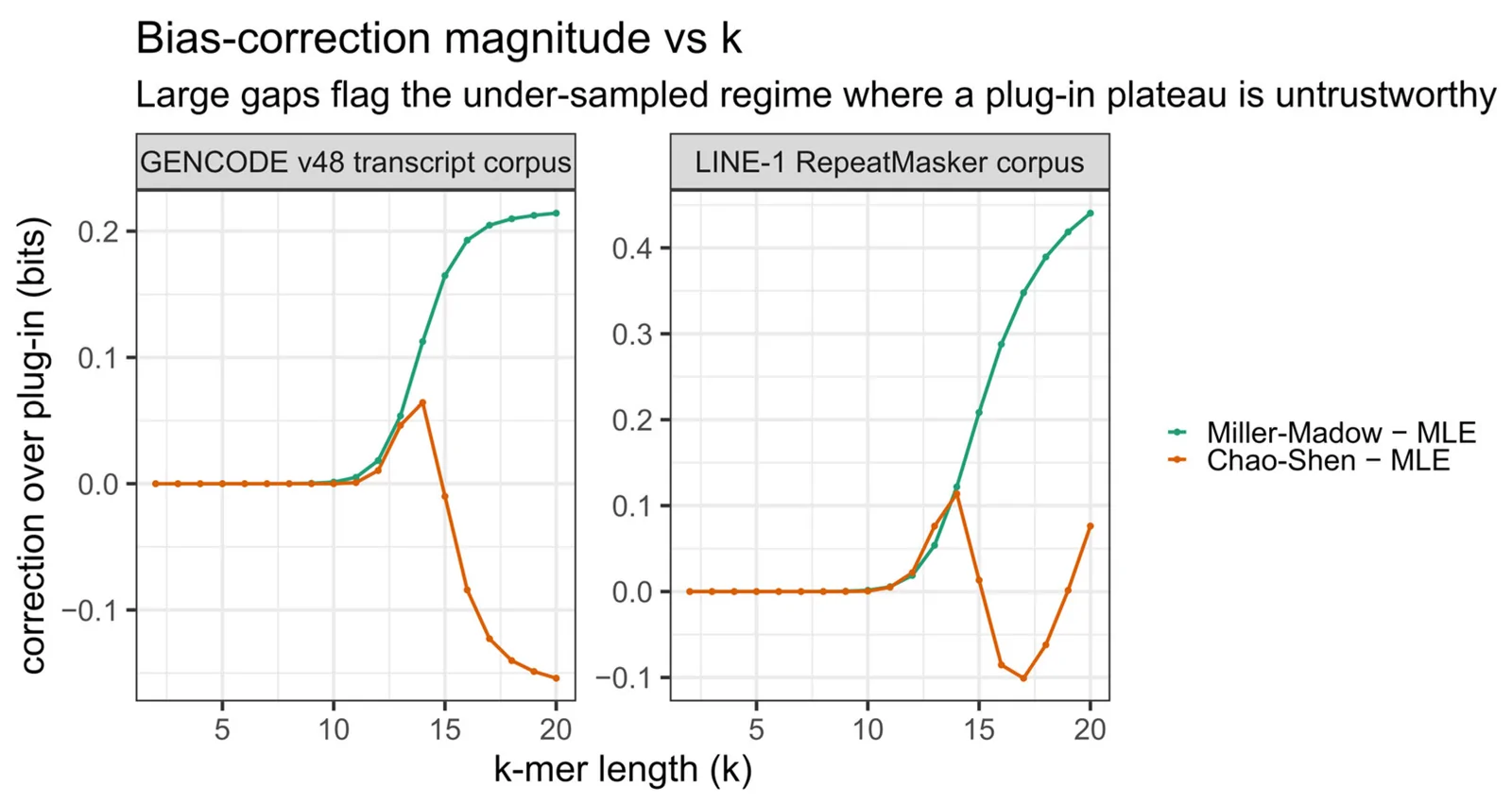
Bias-correction magnitude of the k-mer entropy estimators. For the GENCODE v48 transcript corpus and the LINE-1 RepeatMasker corpus, the difference between each bias-corrected Shannon-entropy estimator (Miller–Madow and Chao-Shen) and the plug-in maximum-likelihood estimator is shown as a function of k. The corrections are negligible for *k* ≲ 11 and grow with k; the divergent, erratic behavior of the Chao-Shen correction at large k marks the under-sampled regime in which a plug-in entropy plateau would be untrustworthy. Applying these corrections is what keeps the entropy estimates interpretable into the operational *k* = 17 range.

**Figure 4:**
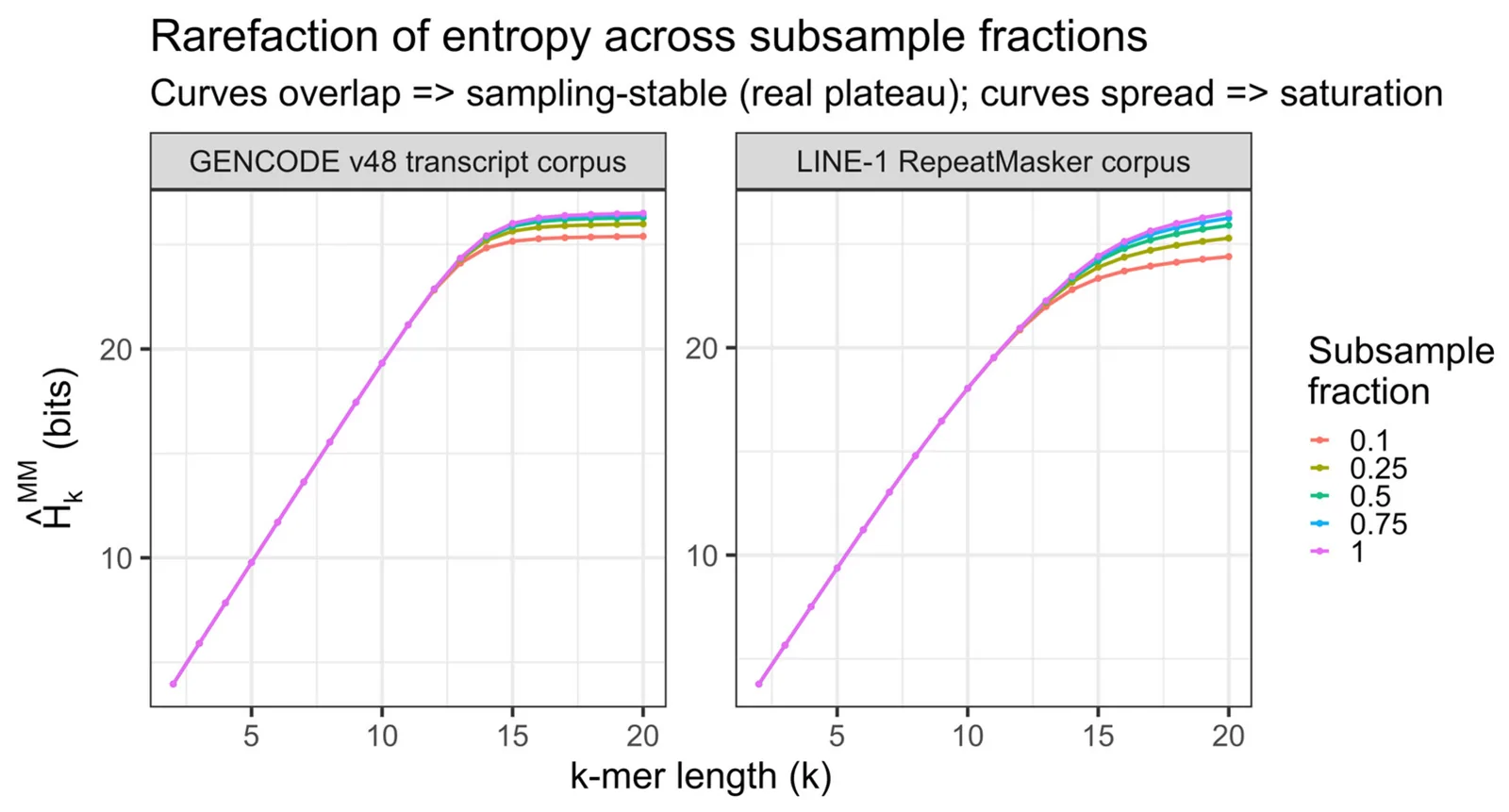
Rarefaction of k-mer entropy across corpus subsample fractions. Miller–Madow entropy is re-estimated after randomly subsampling each corpus to 10–100% of its k-mers. At small to moderate k the curves overlap, indicating that the entropy estimate is sampling-stable and the observed increase reflects genuine information content; beyond *k* ≈ 14 the curves separate by subsample fraction, marking the onset of finite-sample saturation in which larger k is increasingly under-sampled. This saturation check confirms that the entropy plateau is a real feature of the accessible k range rather than an artifact of a single sample size.

**Figure 5:**
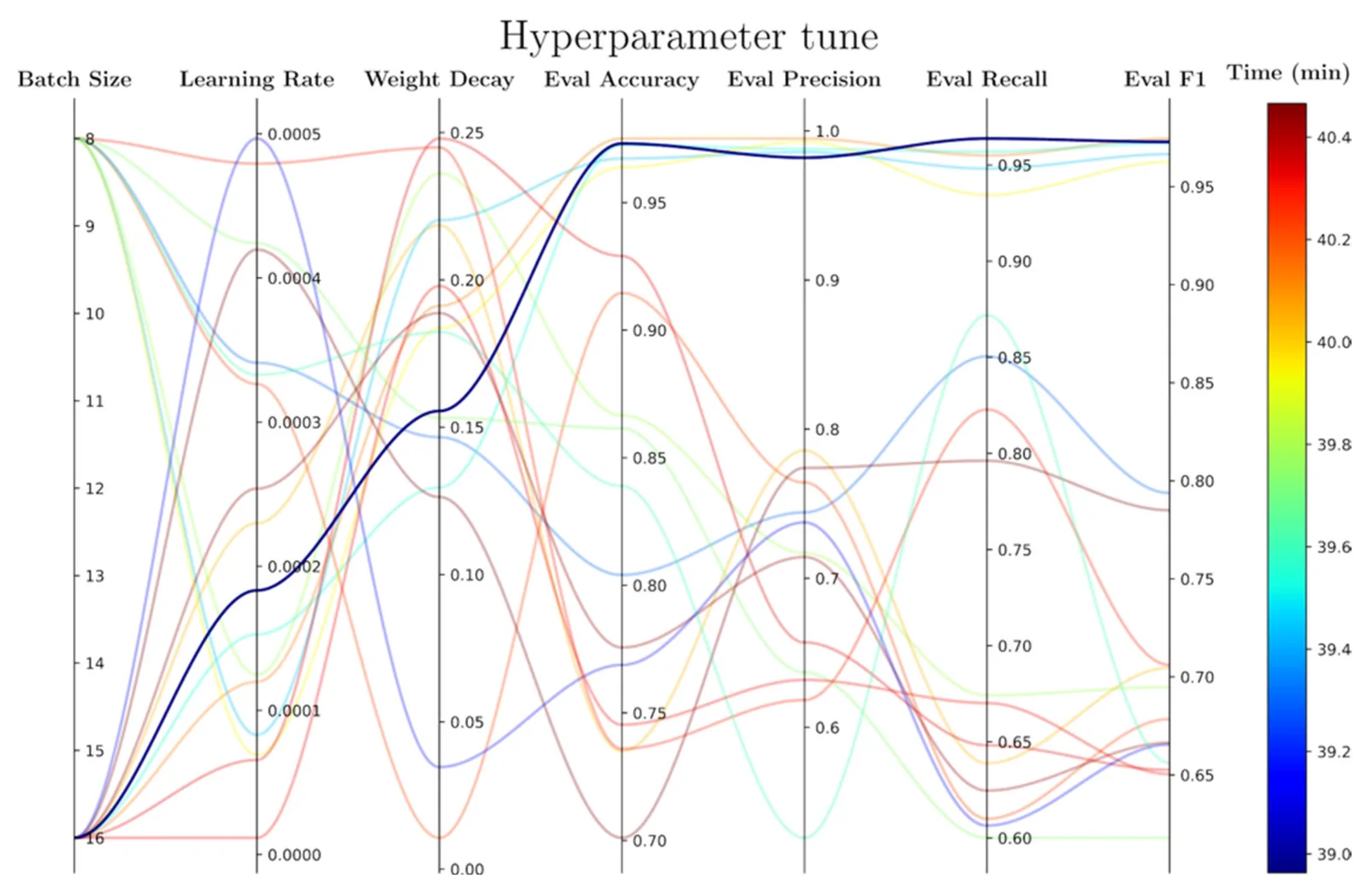
Hyperparameter search for the read-level classifier. Population-Based Training (PBT) was run for 128 trials; the parallel-coordinates plot displays the 16 trials with the shortest training times. Each polyline represents one trial across seven axes: per-device training batch size, the learning rate and weight decay reached through online mutation, and accuracy, precision, recall, and F1 on the held-out set. The color bar denotes the wall-clock runtime of each trial on the same read sample. Runtime therefore represents computational cost and is not expected to correlate with the evaluation metrics. The final configuration was selected primarily by validation F1, with runtime used only as a secondary criterion among trials with comparable F1.

**Figure 6:**
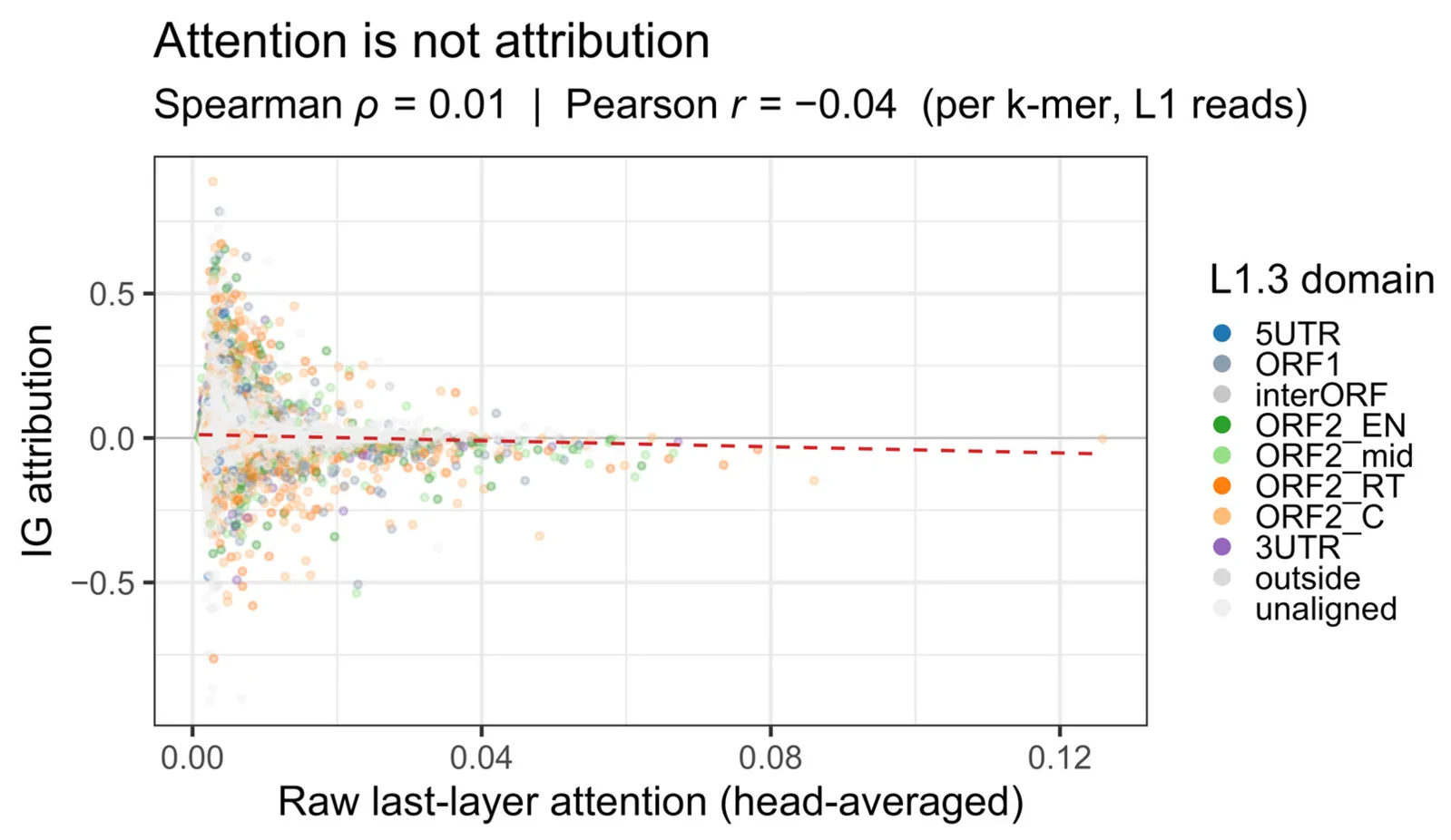
Integrated-gradients attribution is not equivalent to raw attention. For every k-mer in the LINE-1 (L1HS and L1PA) reads, the last-layer head-averaged attention received by the token is plotted against its Layer Integrated Gradients attribution toward the predicted class. The two quantities are essentially uncorrelated (Spearman *ρ* = 0.01, Pearson *r* = −0.04), confirming that raw attention weights are not a reliable proxy for feature importance and supporting the use of gradient-based attribution. Points are colored by the L1.3 consensus domain to which each k-mer aligns.

**Figure 7:**
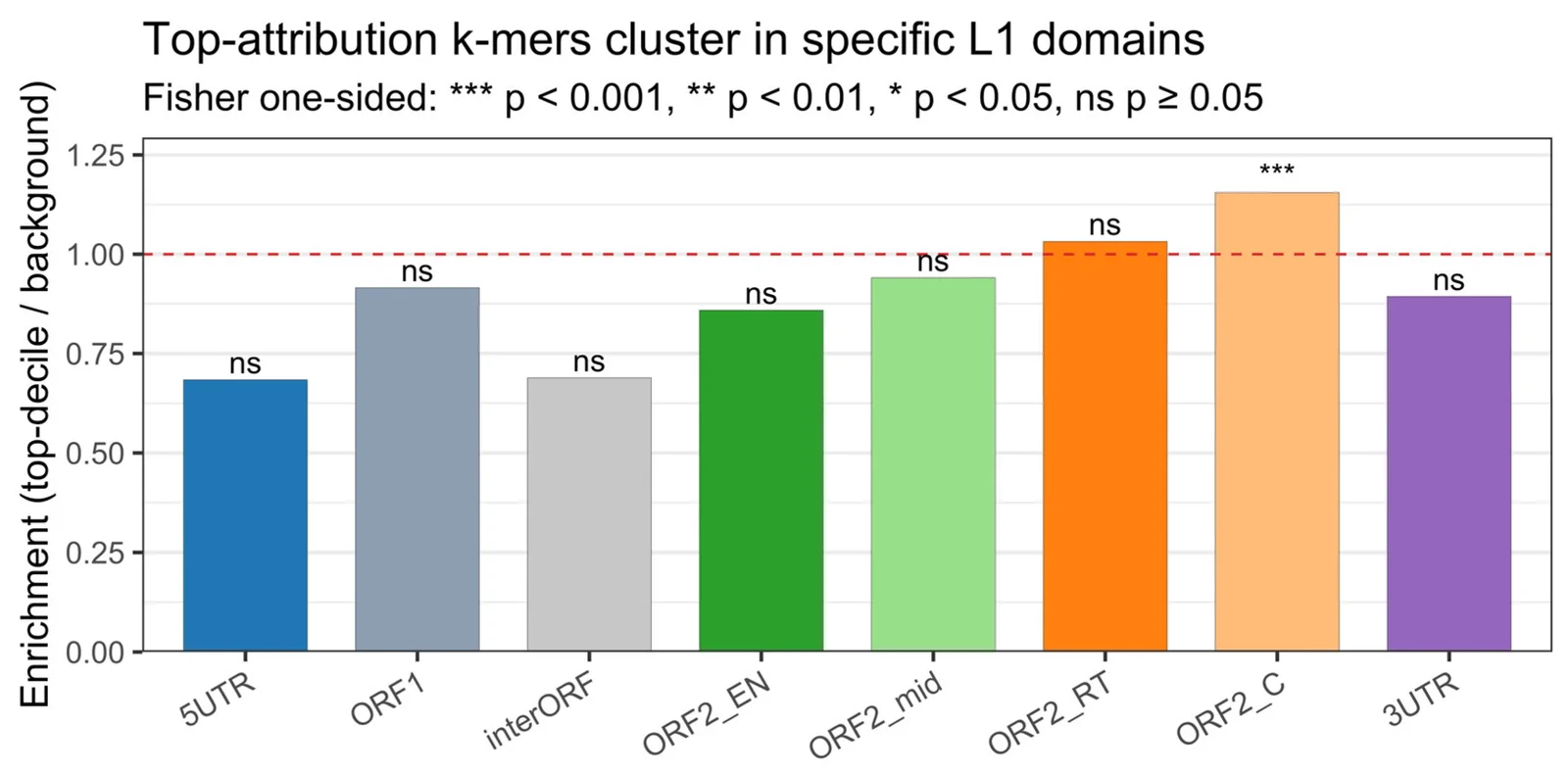
High-attribution k-mers are modestly over-represented in the LINE-1 ORF2 C-terminal domain. Positive-class k-mers were aligned to the L1.3 consensus (GenBank L19088.1) and assigned to annotated LINE-1 domains; for each domain the enrichment of the top attribution decile is shown as the ratio of its observed top-decile fraction to its background fraction. The ORF2 C-terminal domain was modestly over-represented (1.16-fold enrichment; 95% confidence interval, 1.02–1.29, obtained by bootstrap resampling of reads), indicating modest enrichment of high-attribution k-mers within this region.

**Figure 8:**
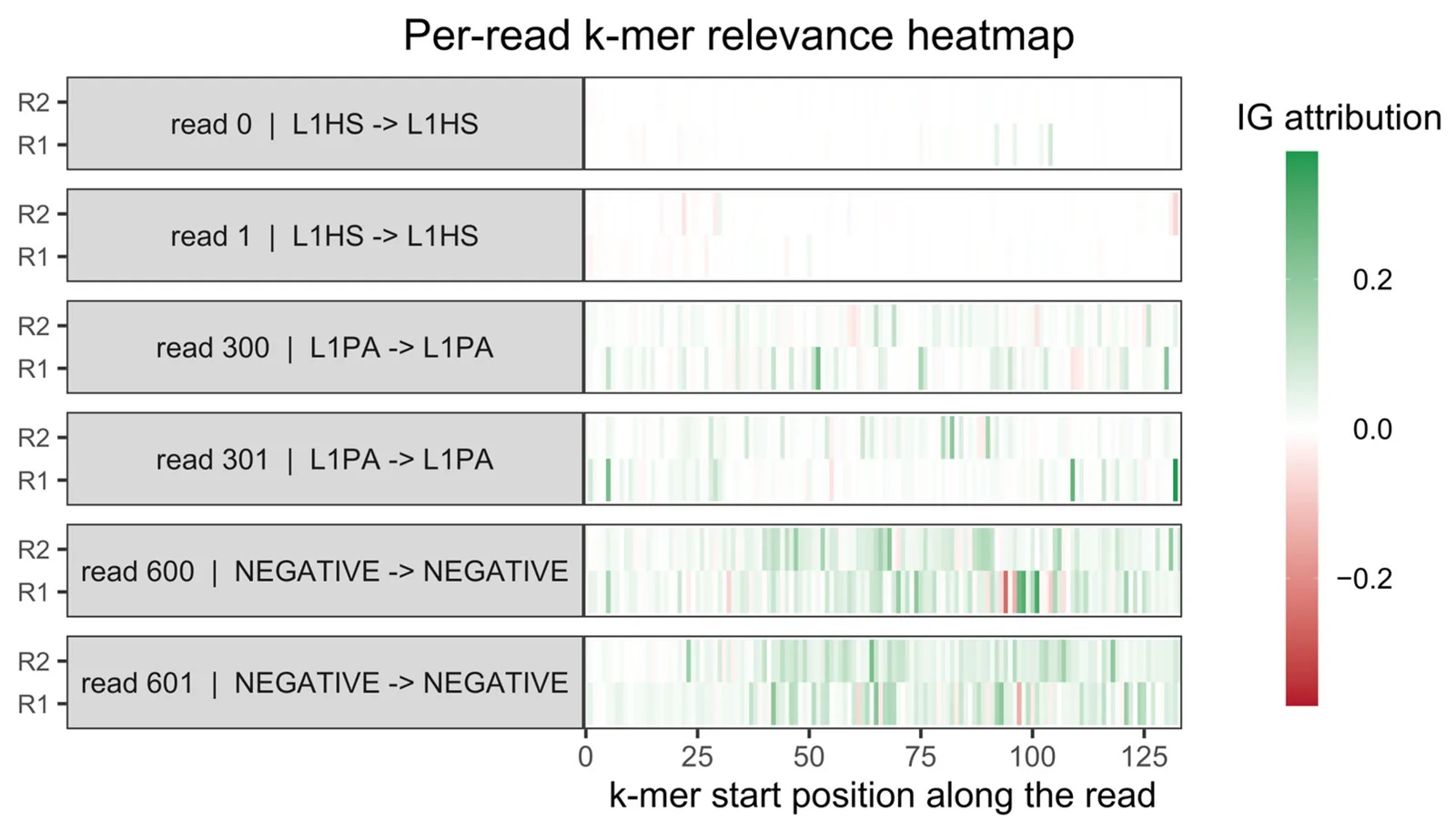
ALBERT relevance for paired-end short reads. Attribution scores were obtained using a k-mer-adapted transformers-interpret workflow. Green shades denote positive contributions toward LINE-1 predictions, while red shades denote negative contributions.

**Figure 9:**
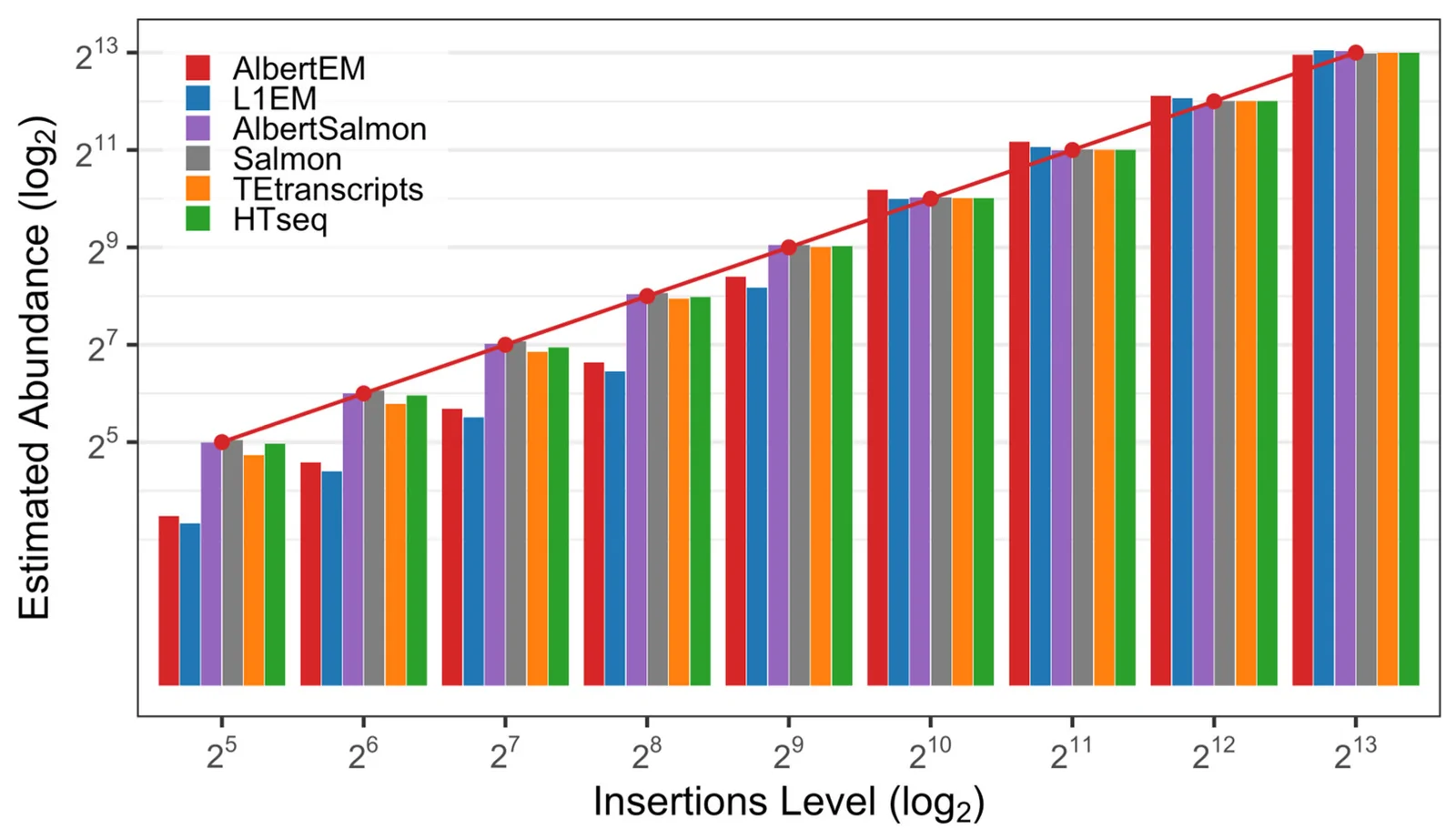
Estimated insertion-equivalent LINE-1 abundance across simulated insertion levels. The *y*-axis shows the summed estimated insertion-equivalent abundance across LINE-1 elements for each simulated dataset. For count-based methods, read counts were normalized by the expected number of reads based on effective LINE-1 length, simulated coverage, and mean fragment length. The red line denotes the expected number of simulated LINE-1 insertions at each insertion level. Values are shown on a logarithmic scale to accommodate the range of simulated insertion numbers.

**Figure 10:**
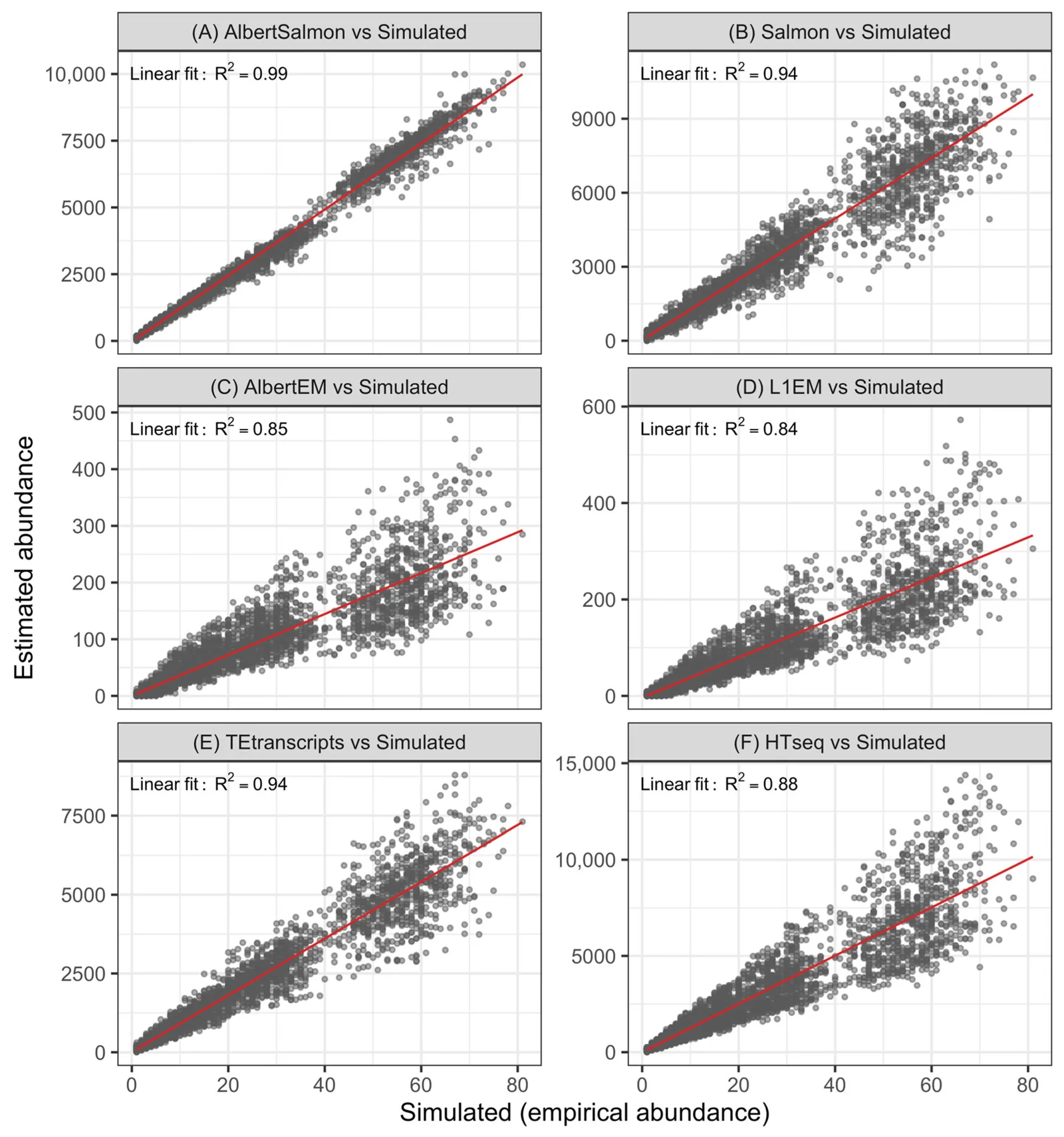
Accuracy of LINE-1 abundance estimates across six computational methods. The calculation was based on the correlation between estimated and expected LINE-1 abundance values. (**A**) AlbertSalmon ranked first in all nine insertion-level strata and all five deletion-probability strata. Its pooled per-element *R*^2^ was 0.99 across the full synthetic grid, while its mean across insertion-level strata was 0.947 ± 0.047 (range 0.865–0.991), representing the smallest dispersion among the methods tested. (**B**–**F**) Salmon, AlbertEM, L1EM, TEtranscripts, and HTSeq showed lower accuracy. Insertion load separated the methods more strongly than deletion probability: from 2^5^ to 2^13^ insertions their *R*^2^ declined by 0.114 for AlbertSalmon and by 0.316–0.622 for the other methods, whereas deletion probability changed *R*^2^ by no more than 0.014 for any method. The red line represents the linear regression model between estimated and expected abundance.

**Figure 11:**
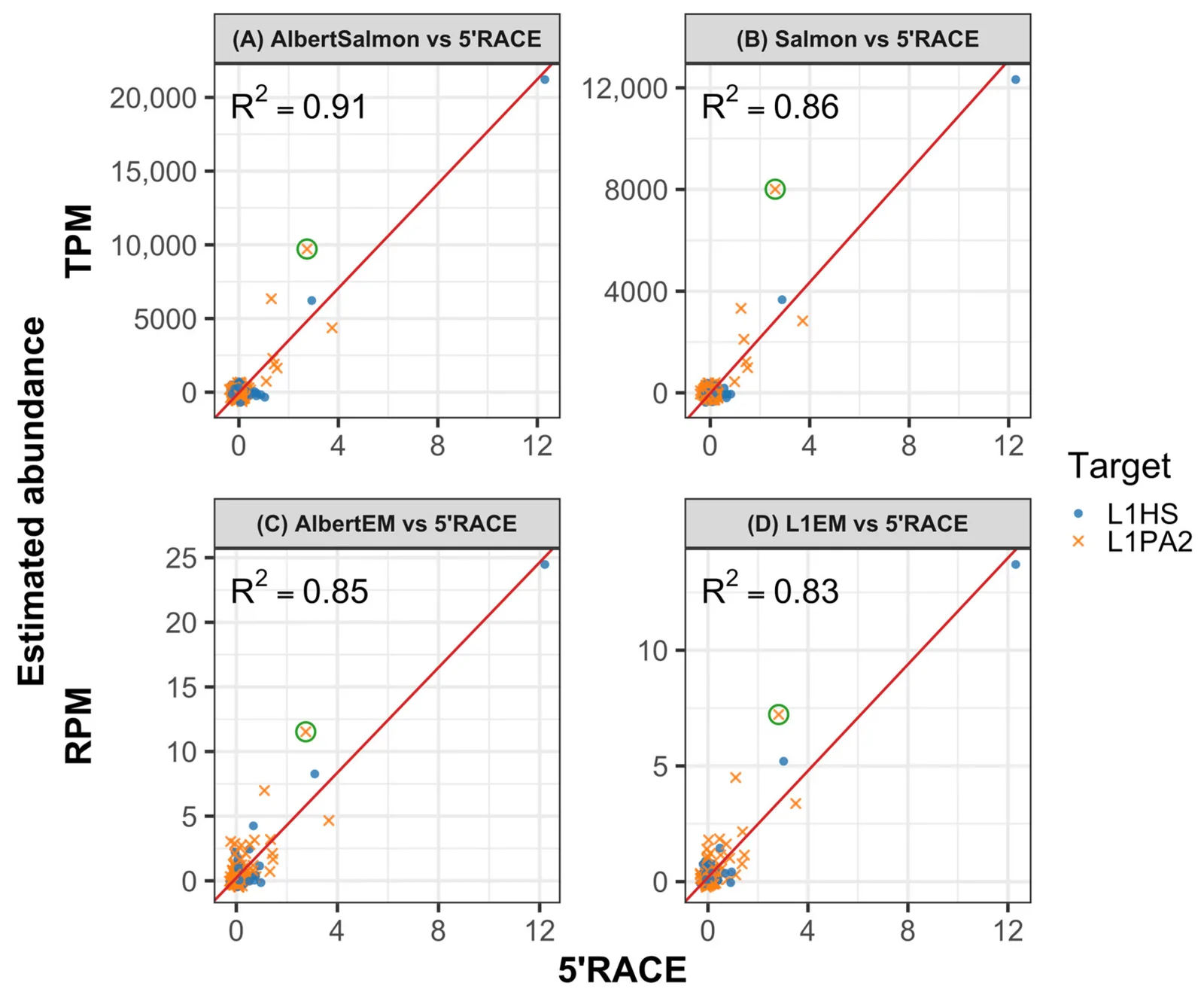
Locus-level comparison of LINE-1 abundance estimates in HEK293 cells using short-read RNA-seq and 5′RACE long-read data. Each point represents an individual RepeatMasker-annotated LINE-1 locus retained after filtering for full-length or near-full-length status based on its genomic annotation, rather than on the physical extent of an individual 5′RACE read. The *x*-axis shows the locus-specific 5′RACE reference signal, expressed as the percentage of the total 5′RACE LINE-1 signal assigned to each locus, derived from approximately 1237 bp 5′ end cDNA products amplified with a LINE-1-specific primer. Short-read abundance estimates were paired with the 5′RACE signal for the corresponding annotated locus. All reported *R*^2^ values were calculated across these individual loci. Most loci cluster near the origin because the 5′RACE reference signal is highly right skewed: one L1HS locus accounts for 16.5% of the total signal carried by the 998 detected loci and the top three loci for 25.4%, a few further L1HS and L1PA2 loci show moderate activity, and the remaining loci have low abundance. The green-circled point marked the L1PA2 locus with the largest residual from the fitted regression. ALBERT-based preprocessing produced greater overall locus-level concordance than Salmon alone (*R*^2^ = 0.91 versus 0.86). These regressions measured concordance with 5′ transcription initiation at individual loci rather than with total LINE-1 transcript abundance. Salmon-based methods report abundance in transcripts per million (TPM), whereas EM-based methods report estimated read assignments normalized as reads per million mapped reads (RPM). TPM and RPM are not directly comparable; therefore, the regressions measure locus-level concordance with the 5′RACE reference, not agreement in absolute abundance. The red line denotes the fitted linear regression.

**Figure 12:**
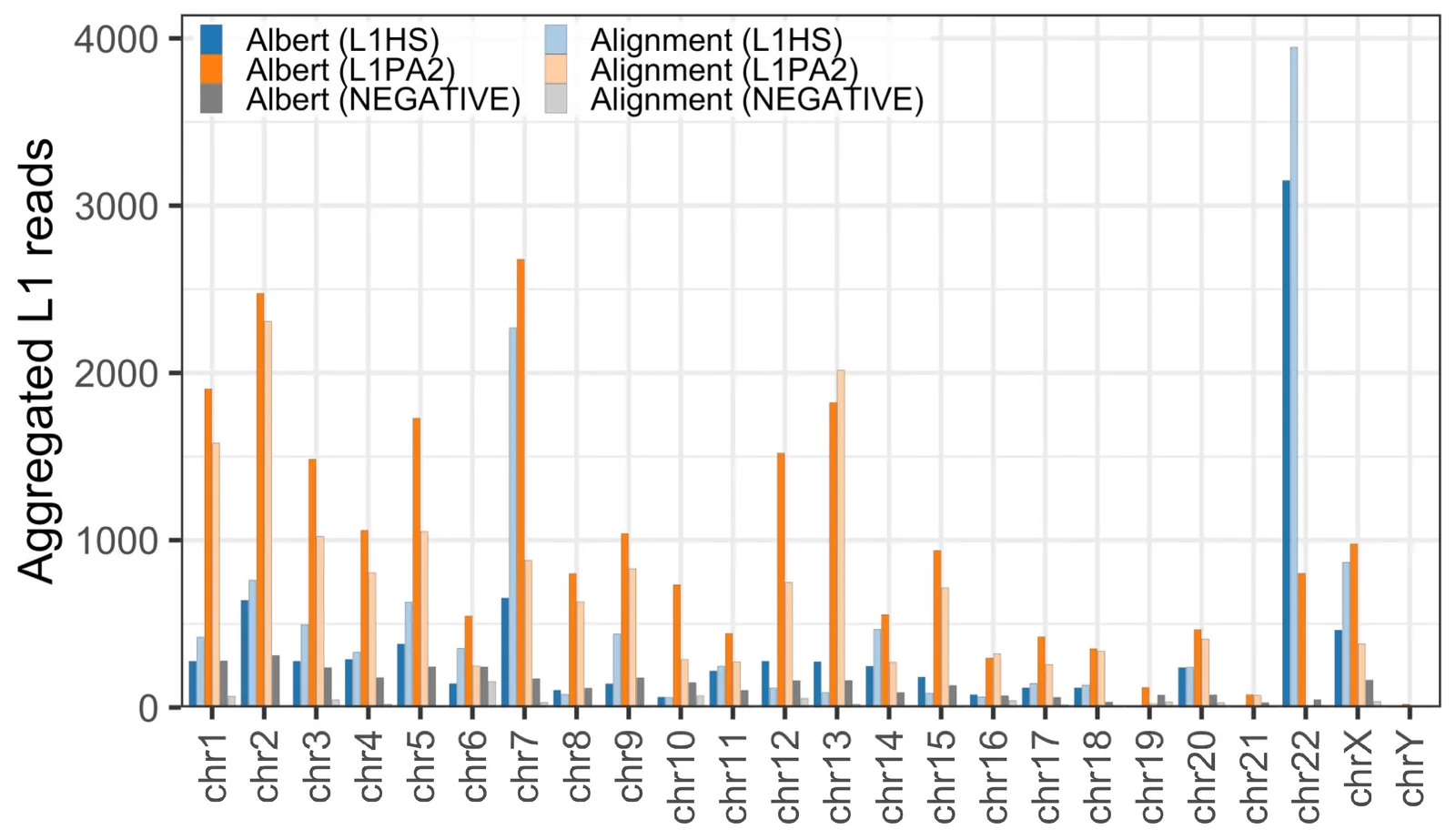
Exploratory chromosome-level comparison of classifier-derived LINE-1 scores and BWA alignment counts in HEK293 5′RACE data. One-hot argmax read counts per class were summed by chromosome and compared with the per-read LINE-1 overlap fraction from the BWA alignment, summed over the same chromosomal references. Because chromosome-level aggregation can be influenced by chromosome size, LINE-1 density, and other broad genomic features, this figure is interpreted as an exploratory concordance assessment rather than as a locus-specific validation.

**Figure 13:**
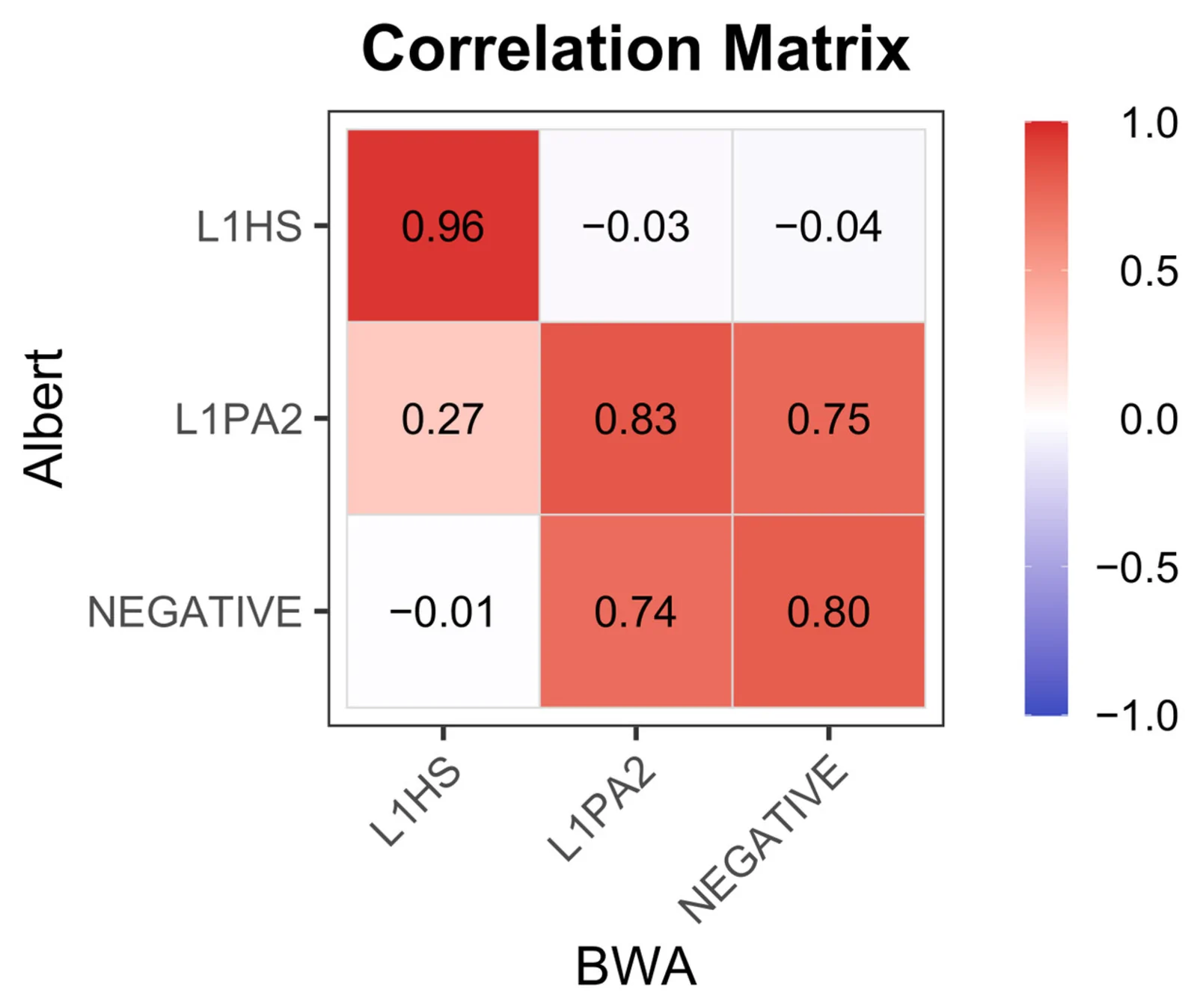
Pearson correlation matrix comparing classifier-derived scores and BWA-derived LINE-1 alignment counts across chromosomes in HEK293 5′RACE data. Correlations were calculated using chromosome-level aggregate values, with the alignment axis restricted to RepeatMasker LINE-1 loci that intersect a full-length L1Base2 element and are at least 6 kb long. The strongest concordance was observed for L1HS, whereas L1PA2 showed moderate agreement. The NEGATIVE category is a classifier rejection class and not a control or a LINE-1 abundance measure. Under the one-hot argmax assignment used here, 38.1% of reads from annotated L1HS elements and 46.3% of reads from annotated L1PA elements in the held-out evaluation set were assigned to NEGATIVE. Correlations involving this category may therefore reflect genuine LINE-1 reads rejected by the classifier. Accordingly, the NEGATIVE comparison is presented as an assessment of assignment composition rather than LINE-1 abundance. These results support class-dependent chromosomal concordance between classifier-derived scores and alignment-based counts, rather than uniform equivalence across all classes.

**Figure 14:**
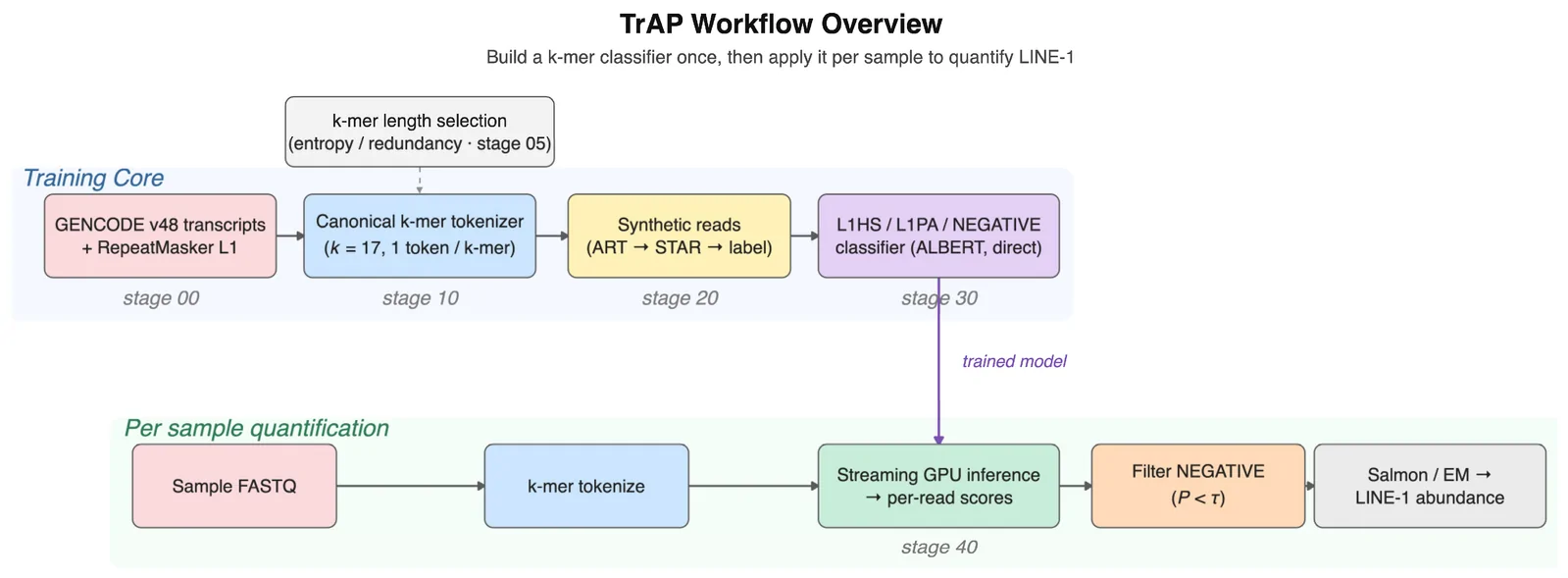
TrAP workflow overview. TrAP is organized into two components that share a canonical k-mer tokenizer. The training component uses GENCODE v48 transcripts and RepeatMasker LINE-1 annotations to generate labeled synthetic paired-end reads through ART simulation and STAR alignment. Each read is encoded using a canonical tokenizer in which every overlapping 17-mer is assigned to one token. The encoded reads are used to train an ALBERT classifier directly on the supervised three-class task of predicting L1HS, L1PA, or NEGATIVE labels. The trained classifier is then applied to each sample: FASTQ reads are k-mer tokenized, classified by GPU-based inference, and filtered using the NEGATIVE-class probability threshold of *τ* = 0.5. Reads with *P*(NEGATIVE) < 0.5 are retained and passed to Salmon- or expectation–maximization-based estimation of LINE-1 abundance.

**Table 1:** Dataset composition for ALBERT classifier training and evaluation. Composition of the simulated transcript-derived read dataset generated from GENCODE v48 and used to train, validate, and test the ALBERT sequence classifier. Reads were labeled as L1HS, L1PA, or NEGATIVE based on overlap with RepeatMasker LINE-1 annotations. L1HS and L1PA together represent LINE-1-positive reads, whereas NEGATIVE denotes reads not assigned to these LINE-1 classes. Dataset splitting was performed at the transcript level so that reads from the same transcript were assigned to only one split.

Split	L1HS	L1PA	NEGATIVE	Total
Training	60,639	2,439,877	2,161,900	4,662,416
Validation	19,641	811,233	723,285	1,554,159
Test (held-out)	19,499	814,814	720,295	1,554,608
Total	99,779	4,065,924	3,605,480	7,771,183

**Table 2:** Classification performance of the ALBERT sequence classifier. Per-class and aggregate performance are shown for the validation split, which was used for checkpoint selection and early stopping. Metrics include F1 score, precision, recall, PR-AUC, and ROC-AUC for each class, together with macro-averaged performance across classes. Overall accuracy (0.959), Cohen’s kappa (0.920), and Matthew’s correlation coefficient (0.920) summarize overall classifier performance.

Metric	L1HS	L1PA	NEGATIVE	Macro Avg
F1	0.670	0.960	0.965	0.865
Precision	0.723	0.979	0.944	0.882
Recall	0.624	0.942	0.987	0.851
PR-AUC	0.696	0.988	0.979	0.888
ROC-AUC	0.978	0.985	0.988	0.984
N	19,641	811,233	723,285	1,554,159

**Table 3:** Comparator tools used for LINE-1 abundance estimation. HTSeq and TEtranscripts were used as independent alignment-based baselines, while Salmon and the EM-based estimator were also evaluated after ALBERT-based preprocessing because their probabilistic assignment frameworks are more directly compatible with transformer-assisted read-level discrimination.

Tool	Purpose	Assumptions	Key Features
HTSeq	Counts reads mapped to genomic features (annotated LINE-1 in this comparison).	Assumes accurate alignment and annotation; does not model multi-mapping reads explicitly.	Simple, widely used for RNA-seq quantification.
TEtranscripts	Quantifies transposable element expression alongside gene expression.	Assumes TE annotations are correct; uses multi-mapping handling for repetitive elements.	Integrates TE and gene quantification.
Salmon	Provides fast, bias-aware annotation quantification using quasi-mapping.	Assumes reference annotations are accurate; probabilistic assignment of multi-mapped reads.	Lightweight, fast; outputs TPM and read counts; bias correction.
L1EM	Estimates locus-specific LINE-1 RNA abundance using an EM algorithm.	Assumes RepeatMasker annotations; models multi-mapping reads probabilistically.	Locus-level resolution; EM-based iterative refinement; tailored for LINE-1 quantification.

**Table 4:** Per-stratum accuracy of LINE-1 abundance estimates across the nine synthetic insertion-level strata. Each row summarizes the per-element coefficient of determination obtained by one method across insertion-level strata spanning 32 to 8192 inserted elements in powers of two. Δ*R*^2^ is the mean paired difference between AlbertSalmon and the indicated comparator, calculated within strata; positive values favor AlbertSalmon. Two-sided paired Wilcoxon signed-rank tests across the nine strata yielded *p* = 0.0039 for every comparison, the minimum attainable two-sided value at *n* = 9 and indicates that AlbertSalmon ranked first in every paired stratum. Paired bootstrap analysis over strata using 5000 resamples produced a mean difference of +0.209 (95% CI, 0.122–0.319) for AlbertSalmon versus Salmon. In a separate comparison, the mean difference between AlbertEM and L1EM was +0.016 (95% CI, 0.005–0.024). The resampling unit was a design stratum rather than an independent replicate.

Method	Mean *R*^2^ ± SD	Range	Δ*R*^2^
AlbertSalmon	0.947 ± 0.047	0.865–0.991	—
Salmon	0.739 ± 0.206	0.310–0.919	+0.209
TEtranscripts	0.697 ± 0.230	0.304–0.926	+0.251
HTSeq	0.630 ± 0.249	0.246–0.884	+0.317
AlbertEM	0.398 ± 0.116	0.202–0.519	+0.550
L1EM	0.382 ± 0.123	0.187–0.534	+0.565

**Table 5:** Sensitivity of locus-level concordance with the 5′RACE reference. Each row reports the per-locus Pearson *R*^2^ against the 5′RACE reference signal for all four methods scored on identical loci, together with the paired within-quantifier differences: AlbertSalmon minus Salmon and AlbertEM minus L1EM. The matched set comprised the loci with a finite estimate from all four methods and non-zero 5′RACE signal. Leave-one-out analyses excluded the dominant L1HS locus, the largest-residual L1PA2 locus, or both to distinguish regression leverage from residual magnitude. Across the full matched set, Spearman *ρ* was 0.449 for AlbertSalmon, 0.425 for Salmon, 0.459 for AlbertEM, and 0.448 for L1EM. Paired bootstrap 95% confidence intervals for Δ*R*^2^, based on 5000 resamples, included zero for both within-quantifier comparisons.

Set Variant	Set Size	Albert Salmon	Salmon	Albert Em	L1EM	Δ*R*^2^ Salmon Pair	Δ*R*^2^ EM Pair
All-matched loci	156	0.863	0.783	0.873	0.841	+0.080	+0.033
Restricted to full-length L1Base2	136	0.869	0.797	0.877	0.845	+0.072	+0.032
Excluding the largest-residual locus	155	0.886	0.841	0.895	0.875	+0.045	+0.020
Excluding the dominant L1HS locus	155	0.591	0.532	0.633	0.618	+0.058	+0.015
Excluding both	154	0.515	0.455	0.567	0.553	+0.060	+0.014

## Data Availability

The datasets analyzed in this study are available from the NCBI Sequence Read Archive under accession numbers SRR3997504 and SRR4099955. The TrAP source code, analysis scripts, and supporting materials are publicly available at https://github.com/andchamorro/TrAP (version 1.0.0) (accessed on 21 September 2026) and Zenodo (https://doi.org/10.5281/zenodo.22757448). To support reproducibility, each pipeline stage records a run manifest capturing software versions, random seeds, tool parameters, and dataset checksums, and reference inputs are verified against fixed checksums so that analyses can be reproduced from their recorded outputs.

## Supplementary Materials

The following supporting information can be downloaded at https://www.mdpi.com/article/10.3390/ijms27219762/s1.
