# Supplementary material for: A Transformers Analysis Pipeline (TrAP) to Evaluate LINE-1 RNA Sequence Content
Source: Int J Mol Sci. 2026 Oct 30;27(21):9762. doi: 10.3390/ijms27219762 (PMC13762667; doi:10.3390/ijms27219762)
Supplement: Supplementary file 1 [file ijms-27-09762-s001.zip › ijms-4595857-supplementary.pdf]

Article

Supplementary Materials: A Transformers Analysis Pipeline (TrAP) to Evaluate LINE-1 RNA Sequence Content

Andres D. Chamorro-Parejo, Kenneth S. Ramos

Table S1. Held-out test-split performance and confusion matrix. Rows show the true class and columns the predicted class under argmax assignment. Row totals correspond to 19,499 L1HS, 814,814 L1PA, and 720,295 NEGATIVE read pairs. The rightmost columns report per-class precision, recall, and F1. Aggregate performance was 0.9589 accuracy, 0.8604 macro-averaged F1, 0.9195 Cohen's kappa, 0.9203 Matthews correlation coefficient, 0.9856 macro ROC–AUC, and 0.8844 macro PR–AUC. The test split was reserved during model development and was not used for checkpoint selection or early stopping.

True class	Predicted L1HS	Predicted L1PA	Predicted NEGATIVE	Total	Precision	Recall	F1
L1HS	11,988	7486	25	19,499	0.703	0.615	0.656
L1PA	5061	767,643	42,110	814,814	0.979	0.942	0.960
NEGATIVE	8	9233	711,054	720,295	0.944	0.987	0.965

Table S2. Coefficient of determination by insertion level. Per-stratum Pearson R^2 between estimated and expected per-element abundance for each method across the nine insertion-level strata of the synthetic chromosome 1 benchmark. Column headings indicate the insertion level as the exponent n in 2^n. The final columns report the mean ± standard deviation, range, and number of strata in which each method achieved the highest R^2.

Method	2^5	2^6	2^7	2^8	2^9	2^{10}	2^{11}	2^{12}	2^{13}	Mean ± SD	Range	Strata won
AlbertSalmon	0.991	0.967	0.985	0.983	0.975	0.950	0.933	0.865	0.877	0.947 ± 0.047	0.865–0.991	9
Salmon	0.771	0.837	0.901	0.919	0.896	0.810	0.704	0.501	0.310	0.739 ± 0.206	0.310–0.919	0
AlbertEM	0.519	0.460	0.462	0.505	0.386	0.461	0.357	0.225	0.202	0.398 ± 0.116	0.202–0.519	0
L1EM	0.534	0.452	0.434	0.489	0.384	0.435	0.327	0.197	0.187	0.382 ± 0.123	0.187–0.534	0
TEtranscripts	0.926	0.830	0.891	0.852	0.802	0.717	0.585	0.363	0.304	0.697 ± 0.230	0.304–0.926	0
HTSeq	0.864	0.884	0.831	0.793	0.729	0.581	0.462	0.280	0.246	0.630 ± 0.249	0.246–0.884	0

Table S3. Coefficient of determination by deletion probability. Per-stratum Pearson R^2 between estimated and expected per-element abundance for each method across five deletion-probability strata of the synthetic chromosome 1 benchmark, spanning 0 to 0.1 in increments of 0.025. All methods were evaluated on the same simulated libraries, with each stratum analyzed separately. The final columns report the mean ± standard deviation, range, and number of strata in which each method achieved the highest R^2. Values are reported to four decimal places because R^2 varied by no more than 0.014 across the deletion-probability range for any method.

Method	0.000	0.025	0.050	0.075	0.100	Mean ± SD	Range	Strata won
AlbertSalmon	0.9942	0.9941	0.9939	0.9943	0.9941	0.9941 ± 0.0001	0.9939–0.9943	5
Salmon	0.9530	0.9520	0.9522	0.9501	0.9518	0.9518 ± 0.0011	0.9501–0.9530	0
AlbertEM	0.8677	0.8642	0.8678	0.8679	0.8603	0.8656 ± 0.0034	0.8603–0.8679	0
L1EM	0.8584	0.8532	0.8576	0.8571	0.8444	0.8542 ± 0.0058	0.8444–0.8584	0
TEtranscripts	0.9511	0.9537	0.9521	0.9530	0.9571	0.9534 ± 0.0023	0.9511–0.9571	0
HTSeq	0.8982	0.9021	0.8982	0.9002	0.8991	0.8995 ± 0.0016	0.8982–0.9021	0

Table S4. Paired comparisons across strata. Each row compares AlbertSalmon with one comparator or a TrAP configuration with its corresponding quantifier, using the paired per-stratum R^2 values reported in Tables S2 and S3. ΔR^2 is the mean paired difference across strata, with 95% confidence intervals obtained from 5000 paired bootstrap resamples. The p values were obtained from two-sided paired Wilcoxon signed-rank tests. A p value equal to the minimum attainable value for the number of strata indicates complete directional consistency rather than the magnitude of the differences.

Axis	Comparison	Number of strata	Strata won	Stratum lost	Mean ΔR^2	Min ΔR^2	95% CI	Wilcoxon p	p floor
Insertion level	AlbertSalmon vs AlbertEM	9	9	–	+0.550	+0.472	0.507 to 0.598	0.0039	0.0039
Insertion level	AlbertSalmon vs HTSeq	9	9	–	+0.317	+0.083	0.196 to 0.450	0.0039	0.0039
Insertion level	AlbertSalmon vs L1EM	9	9	–	+0.565	+0.457	0.518 to 0.616	0.0039	0.0039
Insertion level	AlbertSalmon vs Salmon	9	9	–	+0.209	+0.063	0.122 to 0.319	0.0039	0.0039
Insertion level	AlbertSalmon vs TEtranscripts	9	9	–	+0.251	+0.065	0.147 to 0.374	0.0039	0.0039
Insertion level	AlbertEM vs L1EM (within quantifier)	9	8	2^5	+0.016	-0.015	0.005 to 0.024	0.0195	0.0039

Deletion probability	AlbertSalmon vs AlbertEM	5	5	–	+0.129	+0.126	0.126 to 0.132	0.0625	0.0625
Deletion probability	AlbertSalmon vs HTSeq	5	5	–	+0.095	+0.092	0.093 to 0.096	0.0625	0.0625
Deletion probability	AlbertSalmon vs L1EM	5	5	–	+0.140	+0.136	0.136 to 0.145	0.0625	0.0625
Deletion probability	AlbertSalmon vs Salmon	5	5	–	+0.042	+0.041	0.042 to 0.043	0.0625	0.0625
Deletion probability	AlbertSalmon vs TEtranscripts	5	5	–	+0.041	+0.037	0.039 to 0.042	0.0625	0.0625
Deletion probability	AlbertEM vs L1EM (within quantifier)	5	5	–	+0.011	+0.009	0.010 to 0.014	0.0625	0.0625

Table S5. Sensitivity to insertion load and deletion probability. R^2 values at the lowest and highest insertion-level and deletion-probability strata, together with the corresponding differences between endpoints. Values correspond to the endpoints of the two simulation axes reported in Tables S2 and S3.

**Axis**	**Method**	**First stratum**	**Last stratum**	R^2 **first**	R^2 **last**	**Decline**
Insertion level	AlbertSalmon	2^5	2^{13}	0.991	0.877	+0.114
Insertion level	Salmon	2^5	2^{13}	0.771	0.310	+0.461
Insertion level	AlbertEM	2^5	2^{13}	0.519	0.202	+0.316
Insertion level	L1EM	2^5	2^{13}	0.534	0.187	+0.347
Insertion level	TEtranscripts	2^5	2^{13}	0.926	0.304	+0.622
Insertion level	HTSeq	2^5	2^{13}	0.864	0.246	+0.619
Deletion probability	AlbertSalmon	0.000	0.100	0.994	0.994	+0.000
Deletion probability	Salmon	0.000	0.100	0.953	0.952	+0.001
Deletion probability	AlbertEM	0.000	0.100	0.868	0.860	+0.007
Deletion probability	L1EM	0.000	0.100	0.858	0.844	+0.014
Deletion probability	TEtranscripts	0.000	0.100	0.951	0.957	-0.006
Deletion probability	HTSeq	0.000	0.100	0.898	0.899	-0.001

Table S6. Sensitivity of the 5′RACE comparison to classification-error corrections. Two correction factors were applied only to the L1HS estimates from the classifier-dependent configurations, and locus-level fits were recomputed for a pre-specified matched set of [136/156] loci. The recall-only factor (1.603) was the reciprocal of the held-out L1HS recall, whereas the net factor (1.159) was the precision-to-recall ratio and accounted for reads assigned to NEGATIVE and discarded before quantification. Classifier-independent comparators were unchanged. The corrected estimates are presented as a sensitivity analysis rather than an abundance correction because aggregate classification metrics do not establish uniform misclassification across loci.

Factor	Method	Classifier-dependent	Corrected loci	R^2
1.000	AlbertSalmon	yes	0	0.863
1.000	Salmon	no	0	0.783
1.000	AlbertEM	yes	0	0.873
1.000	L1EM	no	0	0.841
1.603	AlbertSalmon	yes	38	0.894
1.603	Salmon	no	0	0.783
1.603	AlbertEM	yes	38	0.910
1.603	L1EM	no	0	0.841
1.159	AlbertSalmon	yes	38	0.881
1.159	Salmon	no	0	0.783
1.159	AlbertEM	yes	38	0.893
1.159	L1EM	no	0	0.841

Table S7. Paired within-quantifier margins under classification-error corrections. Each margin compared a classifier-dependent TrAP configuration with its corresponding classifier-independent quantifier across a pre-specified matched set of [136/156] loci. Both paired margins increased under the recall-only and precision-to-recall corrections, indicating that the locus-level advantage was preserved after accounting for the aggregate L1HS recall deficit.

Correction	Comparison	ΔR^2	TrAP configuration higher
published	AlbertSalmon vs Salmon	+0.080	yes
published	AlbertEM vs L1EM	+0.033	yes
recall only	AlbertSalmon vs Salmon	+0.111	yes
recall only	AlbertEM vs L1EM	+0.069	yes
net	AlbertSalmon vs Salmon	+0.098	yes
net	AlbertEM vs L1EM	+0.052	yes

Table S8. Chromosome-level correlations before and after adjustment for chromosome length. Each row compares one classifier output class with one alignment-derived LINE-1 class across the 24 assembled chromosomes of the GRCh38.p14 primary assembly (GCA_000001405.29). Partial correlations were calculated after regressing both variables on chromosome length and correlating the residuals. Adjustment for chromosome length produced the largest attenuation in correlations involving the NEGATIVE class, whereas the L1HS diagonal correlation remained unchanged.

Classifier class	Alignment class	Pearson r	p	Partial r given length	p	Change in r
L1HS	L1HS	+0.956	3.7×10^{-13}	+0.957	2.9×10^{-13}	+0.001
L1HS	L1PA2	-0.031	0.8850	+0.048	0.8226	+0.079
L1HS	NEGATIVE	-0.040	0.8544	+0.076	0.7257	+0.115
L1PA2	L1HS	+0.273	0.1972	+0.454	0.0259	+0.181
L1PA2	L1PA2	+0.831	5.0×10^{-7}	+0.671	0.0003	-0.160
L1PA2	NEGATIVE	+0.754	2.1×10^{-5}	+0.386	0.0623	-0.368
NEGATIVE	L1HS	-0.012	0.9551	+0.093	0.6640	+0.106
NEGATIVE	L1PA2	+0.736	4.2×10^{-5}	+0.408	0.0476	-0.327
NEGATIVE	NEGATIVE	+0.804	2.2×10^{-6}	+0.155	0.4705	-0.650

Table S9. Dependence of chromosome-level measures on chromosome length. Pearson correlations between each aggregated classifier- or alignment-derived measure and chromosome length across the 24 assembled chromosomes of the GRCh38.p14 primary assembly. NEGATIVE-class read counts and both L1PA2 measures correlated with chromosome length, whereas neither L1HS measure did. These relationships explain why chromosome-length adjustment attenuated correlations involving NEGATIVE and L1PA2 but did not alter the L1HS concordance reported in Table S8.

Axis	Class	Pearson r with chromosome length	p
Classifier read count	L1HS	-0.099	0.6445
Classifier read count	L1PA2	+0.734	4.4×10^{-5}
Classifier read count	NEGATIVE	+0.937	1.6×10^{-11}
Alignment signal	L1HS	-0.048	0.8247
Alignment signal	L1PA2	+0.673	0.0003
Alignment signal	NEGATIVE	+0.826	6.8×10^{-7}

Table S10. Leave-one-chromosome-out analysis of the chromosome-level comparison. Each row reports one classifier-class and alignment-class pairing from Figure 13 after removing each of the 24 chromosomes in turn. The most influential chromosome was defined as the chromosome whose removal produced the largest change in the correlation coefficient; the adjacent column reports the coefficient after its removal. The jackknife range gives the range of coefficients across the 24 leave-one-chromosome-out analyses.

Classifier class	Alignment class	Pearson r	Most influential chromosome	r without chromosome	Jackknife range
L1HS	L1HS	+0.956	chr22	+0.840	0.148
L1HS	L1PA2	-0.031	chr22	+0.641	0.753
L1HS	NEGATIVE	-0.040	chr22	+0.613	0.710
L1PA2	L1HS	+0.273	chr22	+0.660	0.540
L1PA2	L1PA2	+0.831	chr7	+0.910	0.120
L1PA2	NEGATIVE	+0.754	chr7	+0.686	0.122
NEGATIVE	L1HS	-0.012	chr22	+0.416	0.471
NEGATIVE	L1PA2	+0.736	chr13	+0.821	0.165
NEGATIVE	NEGATIVE	+0.804	chr7	+0.858	0.086

Table S11. Training-split class frequencies and inverse-frequency weights. Counts represent the reads in the transcript-level training split. Class weights were calculated as $w_c = N/(k \cdot n_c)$, where N is the total number of training reads, n_c is the number of reads in class c, and $k = 3$ is the number of classes. These weights were used in the weighted cross-entropy objective. Their frequency-weighted mean was 1.0000, increasing the relative contribution of the minority L1HS class while preserving the overall loss scale.

Class	Training reads	Frequency	Weight w_c
L1HS	60,639	0.013006	25.6294
L1PA	2,439,877	0.523307	0.6370
NEGATIVE	2,161,900	0.463687	0.7189
**Total**	**4,662,416**	1.000000	**1.0000** (frequency-weighted mean)

Table S12. L1HS-to-L1PA read ratio by split. Read counts and L1HS-to-L1PA ratios are reported for each of the three transcript-level splits. The similar ratio across splits indicates that the class imbalance was consistently represented rather than concentrated in a single partition.

Split	L1HS reads	L1PA reads	L1PA : L1HS	L1HS frequency
Training	60,639	2,439,877	40.2 : 1	0.013006
Validation	19,641	811,233	41.3 : 1	0.012638
Test (held-out)	19,499	814,814	41.8 : 1	0.012543
Total	99,779	4,065,924	40.7 : 1	0.012840

Table S13. Propagation of L1HS classification errors into abundance estimates. Precision and recall are the held-out read-level values for the L1HS class. Because reads assigned to NEGATIVE were discarded before quantification, the relationship between the true L1HS count and the count assigned to L1HS depended on both precision and recall. If T is the true number of L1HS reads and A is the number assigned to L1HS, then T/A = precision/recall.

Correction	Recall	Precision	Factor	Implied L1HS underestimate	Basis
recall only	0.624	0.723	1.603	37.6%	1/recall; ignores the contamination that precision measures
net	0.624	0.723	1.159	13.7%	precision/recall; the factor that recovers T from A
